# Yamaguchi esterification: a key step toward the synthesis of natural products and their analogs—a review

**DOI:** 10.3389/fchem.2024.1477764

**Published:** 2024-10-11

**Authors:** Ramsha Munir, Ameer Fawad Zahoor, Muhammad Naveed Anjum, Asim Mansha, Ali Irfan, Aijaz Rasool Chaudhry, Ahmad Irfan, Katarzyna Kotwica-Mojzych, Mariola Glowacka, Mariusz Mojzych

**Affiliations:** ^1^ Department of Chemistry, Government College University Faisalabad, Faisalabad, Pakistan; ^2^ Department of Applied Chemistry, Government College University Faisalabad, Faisalabad, Pakistan; ^3^ Department of Physics, College of Science, University of Bisha, Bisha, Saudi Arabia; ^4^ Department of Chemistry, College of Science, King Khalid University, Abha, Saudi Arabia; ^5^ Department of Basic Sciences, Department of Histology, Embriology and Cytophysiology, Medical University of Lublin, Lublin, Poland; ^6^ Faculty of Health Sciences Collegium Medicum, The Mazovian Academy in Plock, Płock, Poland

**Keywords:** Yamaguchi esterification, macrolides, terpenoids, polyketides, peptides, metabolites

## Abstract

The Yamaguchi reagent, based on 2,4,6-trichlorobenzoyl chloride (TCBC) and 4-dimethylaminopyridine (DMAP), is an efficient tool for conducting the intermolecular (esterification) reaction between an acid and an alcohol in the presence of a suitable base (Et_3_N or ^*i*^Pr_2_NEt) and solvent (THF, DCM, or toluene). The Yamaguchi protocol is renowned for its ability to efficiently produce a diverse array of functionalized esters, promoting high yields, regioselectivity, and easy handling under mild conditions with short reaction times. Here, the recent utilization of the Yamaguchi reagent was reviewed in the synthesis of various natural products such as macrolides, terpenoids, polyketides, peptides, and metabolites.

## 1 Introduction

Ester linkage is the cornerstone of modern synthetic chemistry for containing carbonyl functionality and the structural part of most of the precursors in the synthesis of medicinally important natural and synthetic compounds (Haslam, 1980). Apart from the pharmaceutical industry, other industries (such as textile, cosmetics, fragrance, pesticides, fungicides, and coatings) are also dependent on the ester linkage-based synthetic intermediates. Therefore, esterification is an eminent conversion reaction that is usually performed between acid chloride and alcohol, acid anhydride and alcohol, or carboxylic acid and alcohol (Khan et al., 2021). With the profound interest in the ester linkage, several methodologies have been developed, and the most common methodologies are the Mitsunobu reaction (Munawar et al., 2022), Fischer esterification (involving a Lewis acid as the catalyst) (Joseph et al., 2005), Steglich esterification [usually takes place in the presence of DCC, 4-dimethylaminopyridine (DMAP), and DCM) (Munawar et al., 2024), and Yamaguchi protocol (Inanaga et al., 1979). Each of these methods have their limitations; for example, Fischer esterification is a slower reaction and provides a low yield of product (Khan et al., 2021), whereas Steglich esterification utilizes toxic carbodiimide (Jordan et al., 2021). Steglich and Yamaguchi’s methods are distinguished for the use of DMAP as a strong nucleophilic base. Among others, Yamaguchi esterification is a leading and beneficial tool for esterification and has gained significance in the regioisomeric synthesis of macrolides and many other natural products (Majhi, 2021).

**FIGURE 1:**
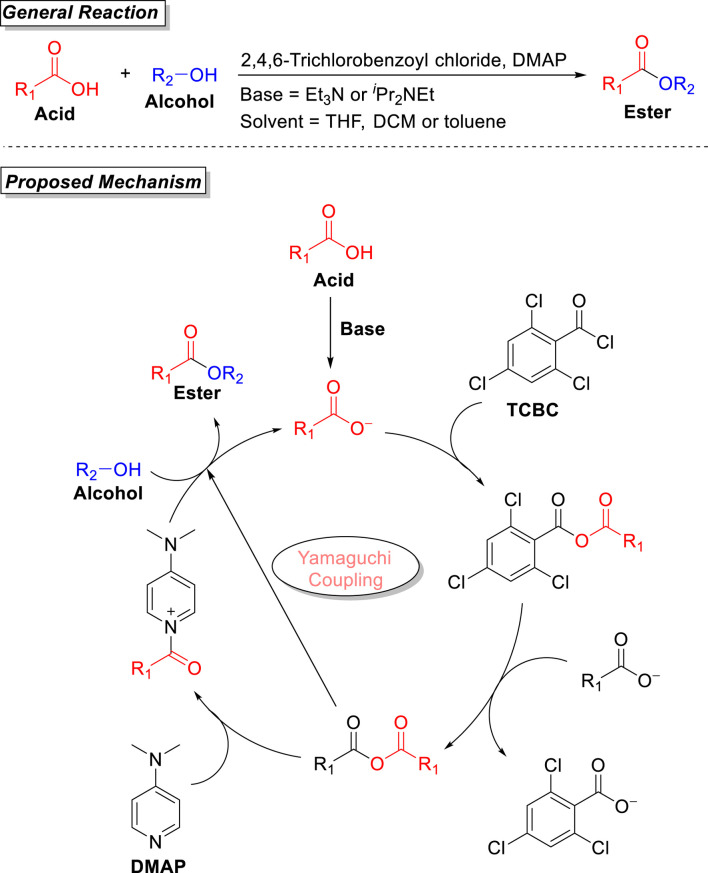
General reaction and proposed mechanism of the Yamaguchi coupling reaction.

The Yamaguchi coupling protocol was first developed by Masaru Yamaguchi et al. (in 1979) during the synthesis of ester (Inanaga et al., 1979). This methodology was mainly based on the reaction between acids and alcohols in the presence of 2,4,6-trichlorobenzoyl chloride (TCBC), with DMAP as the coupling agent and Et_3_N as the base, providing corresponding esters in moderate-to-good yield. Later, this procedure was extended for the synthesis of a variety of macrolactones. The most commonly used solvents are THF, toluene, and DCM to smoothly furnish both primary and secondary esters. Yamaguchi esterification ensures wide substrate scope, mild conditions, and the formation of a regioselective product in moderate-to-good yield. Side by side, Yamaguchi esterification also has disadvantages of less reactivity of TCBC (due to its steric environment), decomposition of the substrates, or poor yield in the case of the total synthesis of very few compounds. Still, this reaction has a wide scope and has been extended to the synthesis of a variety of macrolactones with no epimerization of stereochemistry. The reaction usually takes place either in a single step (direct reaction of carboxylic acid and alcohol) or in two steps (*via* the formation of acid anhydride from carboxylic acid and 2,4,6-trichlorobenzoyl chloride, followed by the attack of alcohol). The two-step method has previously been reported for the synthesis of various lactones of a large ring size, such as 2,4,6-tridemethyl-3-deoymethynolide. The original Yamaguchi procedure was based on a two-step methodology that was later modified as a one-pot (single-step) reaction by Hikota et al. (1990). The detailed mechanistic pathway of this reaction was studied by Dhimitruka and SantaLucia, and the methodology was successfully used for the formation of a Lux-S aspartic acid suppressant. In their investigative studies, benzoyl chloride, *p*-tolyl chloride, and TCBC as an electrophile were used, and the formation of the regioselective product was confirmed only with 2,4,6-trichlorobenzoyl chloride (Yamaguchi reagent), which is the key feature of this esterification reaction (Dhimitruka and SantaLucia, 2006). As shown in Figure 1, the carboxylic acid after deprotonation (*via* the involvement of a base) provides the carboxylate. The coupling of this carboxylate with 2,4,6-trichlorobenzoyl chloride leads to the formation of an anhydride, which, after further coupling with another carboxylate, results in acid anhydride (Park et al., 2022). In the next step, the addition of DMAP results in the formation of pyridinium salt, followed by the nucleophilic attack of the base, which leads toward the formation of the desired ester.

In 2014, a commendable strategy for esterification was introduced by Okuno et al. *via* the use of 2,4,6-trichlorobenzoyl-4-dimethylaminopyridinium chloride as a modified Yamaguchi reagent (Okuno et al., 2014). This TCB-DMAP reagent was prepared (in 90% yield) simply by a reaction between TCBC and DMAP in the presence of THF. It facilitates esterification for broad substrate groups, avoiding the formation of anhydride and can be stored for many years (Yamamoto and Muramatsu, 2019). In 2016, Nishio et al. synthesized a recoverable fluorous Yamaguchi reagent for the efficient esterification of several benzoic acids with alcohols (Nishio et al., 2016) (Supplementary Figure 1).

In addition to esterification and macro-lactonization, the Yamaguchi reagent is much significant for many other organic reactions, especially for the synthesis of carboxylic acid derivatives (Radha Krishna et al., 2022; Mukhopadhyay and Trauner, 2022). For instance, Chandra et al. (2018) utilized their own modified Yamaguchi reagent for amidation, thioesterification, and peptide synthesis. Zulquranain et al. (2020) synthesized pyrazine-2-carboxylic acid derivatives (via the use of the Yamaguchi reagent) to be a cytotoxic agent against *Mycobacterium tuberculosis* (Zulqurnain et al., 2023). In natural product synthesis, the Yamaguchi reagent has been involved in the synthesis of biologically active compounds (Kotammagari, 2014; Wu et al., 2012; Valeev et al., 2019; Molawi et al., 2010), such as stagonolide C (a herbicide isolated from *Cirsium arvense*) (Wu et al., 2012), amphidinolide W (a marine dinoflagellate and a cytotoxic agent against the murine lymphoma cell line with IC_50_ = 3.9 μg/mL) (Shimbo et al., 2002), palmerolide A (exhibits cytotoxicity against melanoma cell line UACC-62 and renal cancer cell line RXF 393) (Pujari et al., 2011), and xyolide (bioactive against *Pythium ultimum*, a plant pathogen) (Maram and Das, 2015) (Supplementary Figure 2). Fascinated by the synthetic utility of the Yamaguchi reagent, Majhi et al. published a review article on the application of Yamaguchi’s method in the synthesis of biologically potent natural products in 2021 (Majhi, 2021). However, an updated compilation of its recent application (2021–2023) in the synthesis of natural products has been presented here.

## 2 Review of the literature

### 2.1 Synthesis of natural macrolides

Esterification and macrolactonization are the commonly involved reactions in the construction of macrolides consisting of simpler to complex frameworks. Here, we present various examples, demonstrating the strong potential of the Yamaguchi reagent in the synthesis of 10–30 (ring size)-membered macrolides.

#### 2.1.1 Synthesis of 10-membered macrolides

##### 2.1.1.1 Reddy’s total synthesis of sumalactone A

**SCHEME 1:**
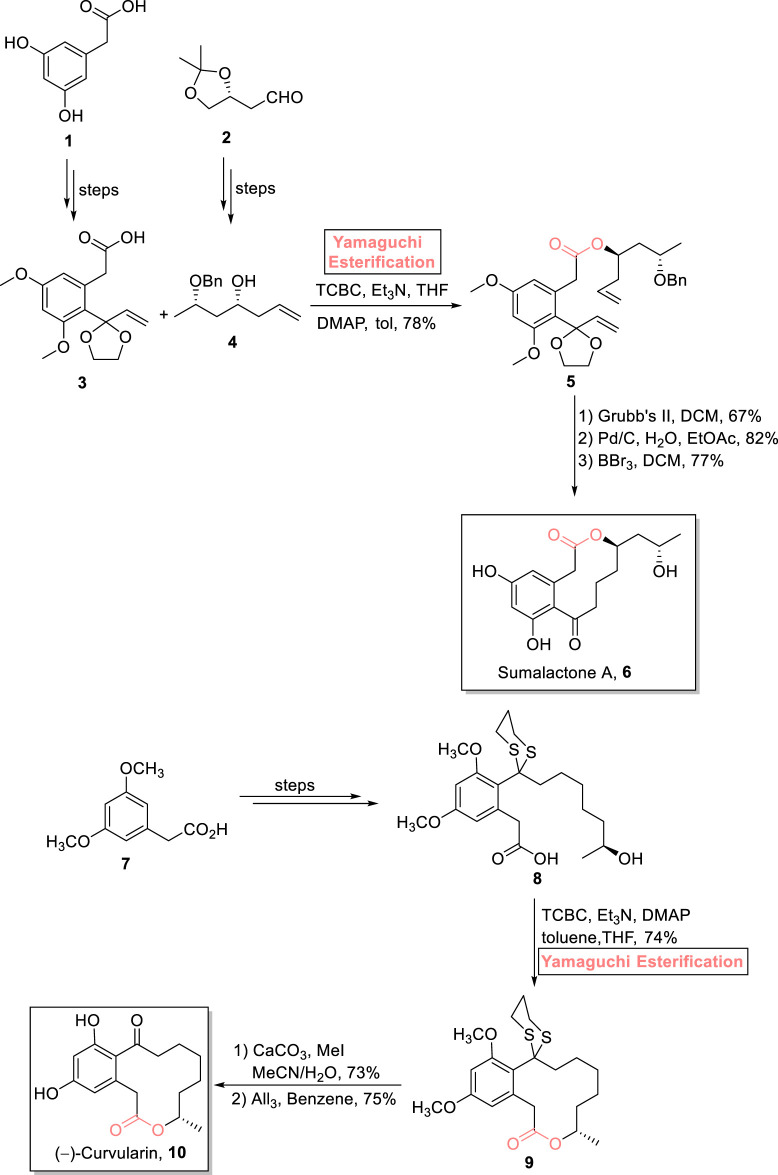
Synthesis of sumalactone A **10** and (−)-curvularin **11**.

Sumalactone A **10** is a 10-membered macrolactone that was isolated from *Penicillium sumatrense*, a marine fungus (Wu et al., 2017). This benzannulated macrolactone is famous for its numerous biological activities, such as anti-fungal, anti-cancer, and anti-inflammatory effects (Scheme 1) (Allu et al., 2019; Shahzadi et al., 2022). As an attractive target of various organic chemists, Reddy et al. (2022) performed the stereoselective synthesis of sumalactone A **10** using easily available inexpensive starting materials **1** and **2** (Reddy et al., 2022). The key steps in their synthetic part involve Yamaguchi esterification with the proper maintenance of stereochemistry of the reacting substrates. As illustrated in the scheme, acid **3** and alcohol **4** were successfully achieved from acid **1** and alcohol **2**, respectively. Both the synthesized compounds, in hand, were subjected to esterification using a Yamaguchi reagent (2,4,6-trichlorobenzoyl chloride) in the presence of Et_3_N, DMAP, and toluene, resulting in high (78%)-yielding ester **5**. Furthermore, Grubb’s second-generation catalyst was used for the RCM reaction of ester **5**, followed by its palladium-catalyzed reduction and deprotection in the presence of BBr_3_ and DCM to finally afford sumalactone A **6** with 77% yield.

#### 2.1.2 Synthesis of 12-membered macrolides

##### 2.1.2.1 Radha Krishna’s total synthesis of (−)-curvularin

One of the 12-membered macrolides, curvularin, is a resorcylic acid lactone that is produced by various fungal sources, that is, *Alternaria*, *Penicillium*, and *Curvularia*. It exhibits diverse biological activities such as cell division prohibition and cytotoxicity against sea urchin embryogenesis (Zhan and Gunatilaka, 2005). With a great deal of interest, Radha Krishna et al. (2022) accomplished its total synthesis using Yamaguchi macrolactonization as a crucial step. For achieving the targeted product, (3,5-dimethoxyphenyl)acetic acid **7** was used as an easily available starting material to build compound **8**. Next, compound **8** was subjected to a well-suited Yamaguchi reagent, leading to the formation of compound **9** with 74% yield. After this, the removal of the 1,3 dithiane group and deprotection of methyl ester groups in ester **9** resulted in the synthesis of (−)-curvularin **10** with 75% yield (Scheme 1).

##### 2.1.2.2 Chambers’s total synthesis of 10-deoxymethynolide

**SCHEME 2:**
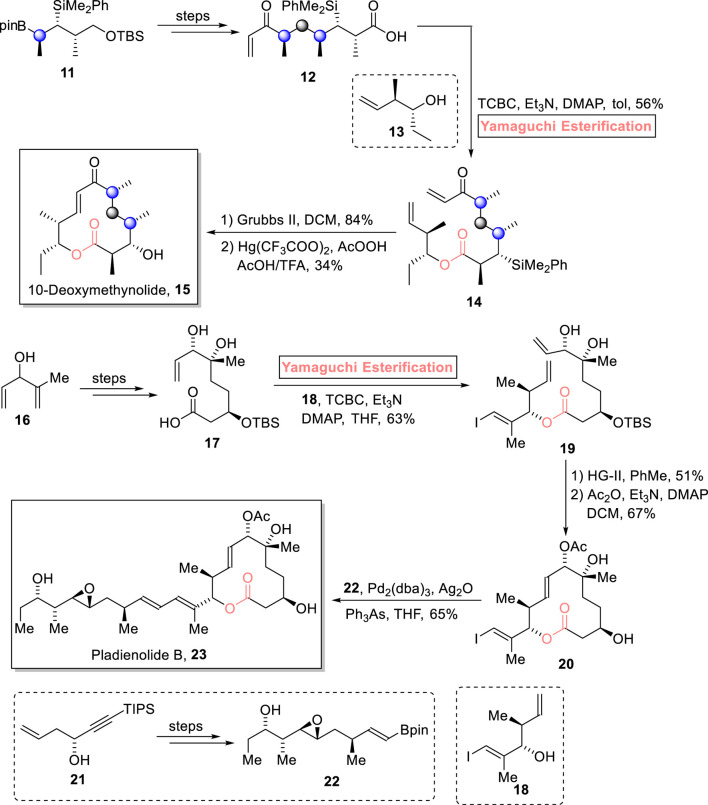
Synthesis of 10-deoxymethynolide **15** and pladienolide B **23**.

Enones are unique synthetic intermediates that have widespread applications in the synthesis of many biologically active natural products. An enone, 10-deoxymethynolide contains a popular polyketide macrolide, having four stereogenic centers, and is expected to be a medicinally important natural product (Wei and Shi, 2013). Chambers et al. (2023) accomplished the efficient 14-step total synthesis of 10-deoxymethynolide **15** by using ester **11** as an easily available enantioenriched starting material (Scheme 2) (Chambers et al., 2023). The settlement of four stereocenters in the target compound was no doubt a challenging task that was successfully made possible using the Yamaguchi protocol. To set the stage for the Yamaguchi esterification protocol, the modification of compound **11** (over a few steps) into compound **12**, followed by treatment with compound **13** in the presence of TCBC, triethyl amine (Et_3_N), DMAP, and toluene, successfully furnished ester **14** with 56% yield. Next, the ring closure reaction of compound **14** using Grubb’s second-generation catalyst and subsequent desilylation completed the total synthesis of 10-deoxymethynolide **15** with 34% yield.

##### 2.1.2.3 Yoo and Krische’s total synthesis of pladienolide B

Pladienolides are 12-membered macrolides isolated from *Streptomyces platensis* in 2004. Owing to their anti-proliferative activity in multi-resistant human tumor cells, this unique family of natural products is gaining prominence as anti-cancer pharmacodynamics in medicinal chemistry (Sakai et al., 2004; Villa et al., 2012). To date, various synthetic reports on the synthesis of pladienolide derivatives have been published. Pioneering this groundbreaking endeavor, Yoo and Krische (2021) devised a robust and economical synthetic route for the synthesis of pladienolide B **23** (consisting of 10 stereogenic centers) in just 10 steps (Scheme 2) (Yoo and Krische, 2021). The salient feature of their synthesis lies in the strategic use of the Yamaguchi method as the leading tool for esterification. To set the stage for Yamaguchi esterification, compound **16** was turned into acid **17** over a few steps. Compound **17**, in hand, was allowed to get esterified with fragment **18** in the presence of TCBC, Et_3_N, DMAP, and THF to result in ester **19** with 63% yield. In the next step, the ring-closing metathesis of compound **19**, followed by acetylation, resulted in compound **20** with 67% yield. Moving toward the final step, compound **20** was made to couple with compound **22** (from compound **21**) under Suzuki conditions to successfully accomplish the target pladienolide B **23** with 65% yield.

#### 2.1.3 Synthesis of 14-membered macrolides

##### 2.1.3.1 Meyer’s total synthesis of amphidinolide R

**SCHEME 3:**
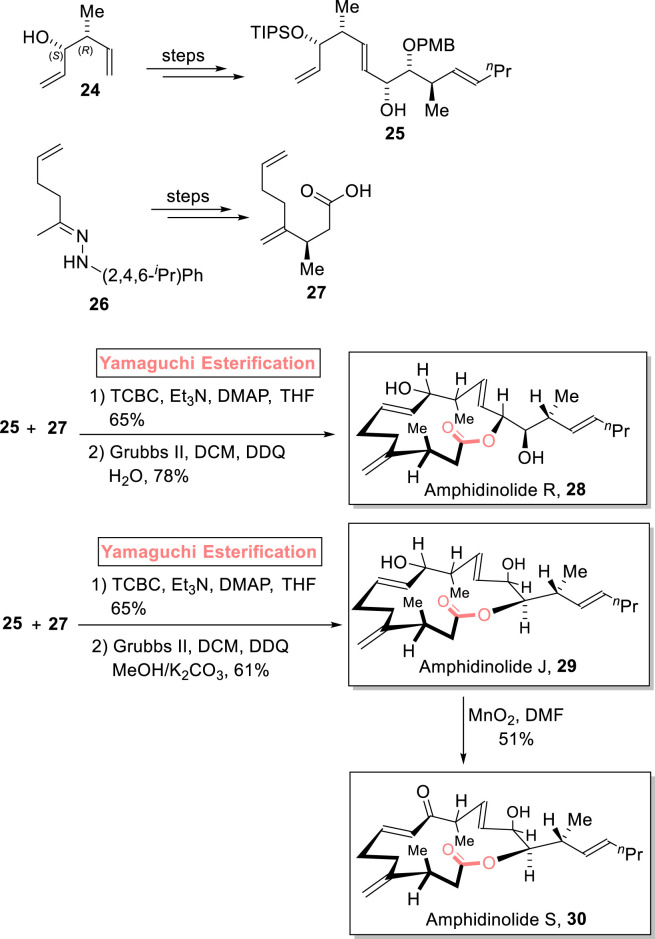
Synthesis of amphidinolide R **28**, amphidinolide J **29**, and amphidinolide S **30**.

Amphidinolides are cytotoxic macrolides, and these were isolated from *Amphidinium* sp. marine dinoflagellates by Kobayashi et al.. Structurally, amphidinolide R is 14-membered, while amphidinolide J and amphidinolide S are 15-membered macrolides (Ishibashi and Kobayashi, 1997; Shotwell and Roush, 2004). Meyer et al. (2023) accomplished the diastereoselective and enantioselective synthesis of amphidinolide R **28** (9 steps) and amphidinolide J **29** (9 steps), along with the first total synthesis of amphidinolide S **30** (10 steps). The achievement of the desired stereochemistry in the products was assured *via* the use of Yamaguchi esterification as a powerful step (Scheme 3) (Meyer et al., 2023). Their methodology involved the independent synthesis of fragments **25** and **27** from compounds **24** and **26**, respectively. Then, fragments **25** and **27** were subjected to esterification using the Yamaguchi protocol in the presence of diethyl amine, TCBC, Et_3_N, and THF. The esterified intermediate was then subjected to Grubb’s second-generation catalyst and DDQ, followed by corresponding work procedures under given conditions to furnish amphidinolide R **28** in 78% yield.

##### 2.1.3.2 Nakazato’s total synthesis of (+)-neopeltolide

**SCHEME 4:**
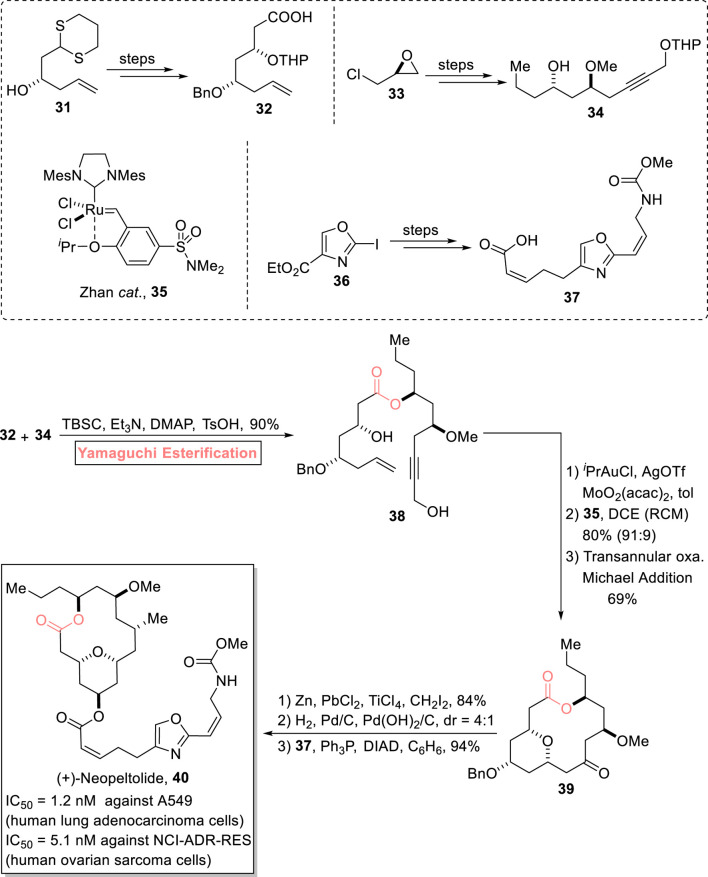
Synthesis of (+)-neopeltolide **40**.

The tetrahydropyran ring is present in most biologically important natural products. One of the tetrahydropyran rings containing a natural product, (+)-neopeltolide **40**, is a 14-membered macrolide, consisting of a lactone ring associated with a 2,4,6-trisubstituted tetrahydropyran scaffold. It was isolated from a deep-water sponge in Jamaica by Wright et al. (2007). It exhibits cytotoxic activity against A549 (human lung adenocarcinoma cells) and NCI-ADR-RES (human ovarian sarcoma cells) with IC_50_ values of 1.2 nM and 5.1 nM, respectively. Furthermore, it also exhibits growth inhibition against *Candida albicans* with a MIC value of 0.62 μg/mL (Ulanovskaya et al., 2008; Bai and Dai, 2015). With these distinctive medicinal features, this scaffold has been the focus of various researchers, and more than 20 reports on its synthesis have been documented. Continuing with the ongoing effort, Nakazato et al. (2022) performed the 11-step total synthesis of (+)-neopeltolide **40** (with 12% overall yield) *via* Yamaguchi esterification of intermediates **32** and **34** as the key step (Scheme 4) (Nakazato et al., 2022). In their synthetic methodology, compounds **32**, **34**, and **37** were prepared from starting materials **31**, **33**, and **36**, respectively. After this, the Yamaguchi esterification of compounds **32** and **34** in the presence of TCBC, Et_3_N, DMAP, and TsOH successfully furnished ester **38** (with 90% yield) as a precursor for the construction of the 14-membered anti-cancer macrolide. Ester **38** then underwent a sequence of Meyer–Schuster rearrangement, Zhan-catalyzed RCM reaction, and Michael addition to result in tetrahydropyran **39** with 69% yield. Next, compound **39** was subjected to Zn-mediated methylenation, hydrogenolysis, and subsequent Mitsunobu coupling with compound **37** to afford the desired natural product **40** with 94% yield.

##### 2.1.3.3 Depa’s total synthesis of neocosmosin A

**SCHEME 5:**
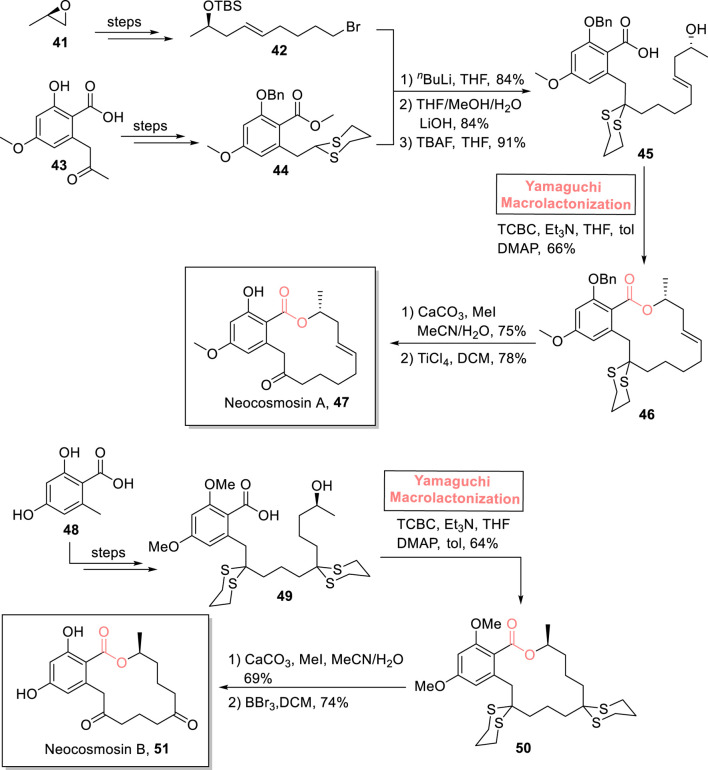
Synthesis of neocosmosin A **47** and neocosmosin B **51**.

Resorcyclic acid lactones are well known in the medicinal world for their remarkable biological profile as they exhibit estrogenic, cytotoxic, nematocidal, anti-viral, and anti-fungal biological activities (Patocka et al., 2013; Hellwig et al., 2003; Abid-Essefi et al., 2004). The 14-membered macrolides, neocosmosin A **47** and neocosmosin B **51,** are resorcyclic acid lactones according to their structural composition. These were isolated in 2012 from *Neocosmospora* sp. of a fungal strain. Neocosmosin A **47** possesses an affinity for binding with human cannabinoid and opioid receptors (Gao et al., 2013). Depa et al. (2021) performed the efficient (14-step) total synthesis of this natural product using propylene oxide **41** and 4-methoxy salicylic acid **43** (as easily available starting materials) with a 4.45% overall yield (Scheme 5) (Depa et al., 2021). The construction of this targeted natural product with the desired stereochemistry entails Yamaguchi macrolactonization as a crucial step. The methodology involved the coupling of bromide **42** and dithiane **44,** followed by hydrolysis and desilylation to furnish hydroxy acid **45** with 91% yield. Next, the Yamaguchi protocol was used for the esterification of acid **45** by treating it with TCBC, Et_3_N, THF, DMAP, and toluene, which resulted in compound **46** with 66% yield. Proceeding toward the last stage of the total synthesis, the dithiane group was removed *via* the treatment of lactone **46** with calcium carbonate and methyl iodide, followed by TiCl_4_-mediated deprotection to successfully afford neocosmosin A **47** with 78% yield.

##### 2.1.3.4 Kumari’s total synthesis of neocosmosin B

Kumari et al. (2022) reported the first total synthesis of neocosmosin B **51** in 12 steps by using Yamaguchi macrolactonization as a key step (Scheme 5) (Kumari et al., 2022). Their synthesis commenced with easily available orsellinic acid **48**, which, over a few steps, provided compound **49**. In the following step, the Yamaguchi reagent was used for the macrolactonization of hydroxyl acid **49** by treating it with TCBC, Et_3_N, THF, and then with DMF and toluene to finally furnish lactone **50** with 64% yield. Finally, the removal of dithiane groups and demethoxylation of lactone **50** successfully furnished the desired neocosmosin B **51** with 74% yield.

##### 2.1.3.5 Dissanayake’s total synthesis of sanctolide A

**SCHEME 6:**
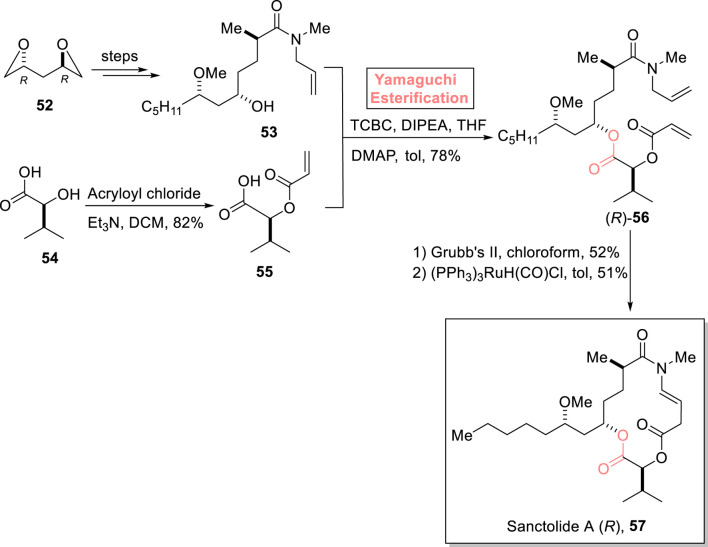
Synthesis of sanctolide A **57**.

Sanctolide A **57** is a 14-membered polyketide and peptide-based macrolide. It was isolated in 2012 by Orjala et al. from *Oscillatoria sancta*, a cyanobacterium (Kang et al., 2012). The structural framework of this hybrid scaffold comprises an *N*-methyl-substituted macrocyclic diester attached with a lipophilic side chain. With these significant structural features, this natural product is expected to show promising pharmaceutical effects. Dissanayake et al. (2023) performed both the total and formal syntheses of sanctolide A **57** by using Yamaguchi esterification as the main step (Scheme 6) (Dissanayake et al., 2023). As shown in Scheme 6, alcohol **53** (synthesized from compound **52** in several steps) was made to react with 2,4,6-trichlorobenzoyl chloride and diisopropylethylamine in THF. Then, carboxylic acid **55** (prepared from the reaction of isovaleric acid **54** and acryloyl chloride) was added to complete the esterification process in the presence of DMAP and toluene, resulting in ester (*R*)-**56** with 78% yield. Hence, the coherence of stereochemistry in the reactants (alcohol **53** and acid **55**) and the product (ester **56**) highlights the success of choosing the Yamaguchi reagent for the esterification process. In the following step, the RCM reaction of ester (*R*)-**56** and the subsequent treatment with (PPh_3_)_3_RuH(CO)Cl furnished the desired sanctolide A (*R*)-**57** with 51% yield.

#### 2.1.4 Synthesis of 15-membered macrolides

##### 2.1.4.1 Meyer’s total synthesis of amphidinolide J and amphidinolide S

As mentioned previously, Meyer et al. (2023) accomplished the synthesis of amphidinolide R **28** and amphidinolide J **29** and the first total synthesis of amphidinolide S **30**. Their simple and facile methodology involved the Yamaguchi esterification of compounds **25** and **27** and subsequent treatment with Grubb’s second-generation catalyst to furnish amphidinolide J **29** with 61% yield. Next, the MnO_2_-induced oxidation of amphidinolide J **29** led to the formation of amphidinolide S **30** with 51% yield (Scheme 3).

##### 2.1.4.2 Lai and Dai’s total synthesis of palmyrolide A

**SCHEME 7:**
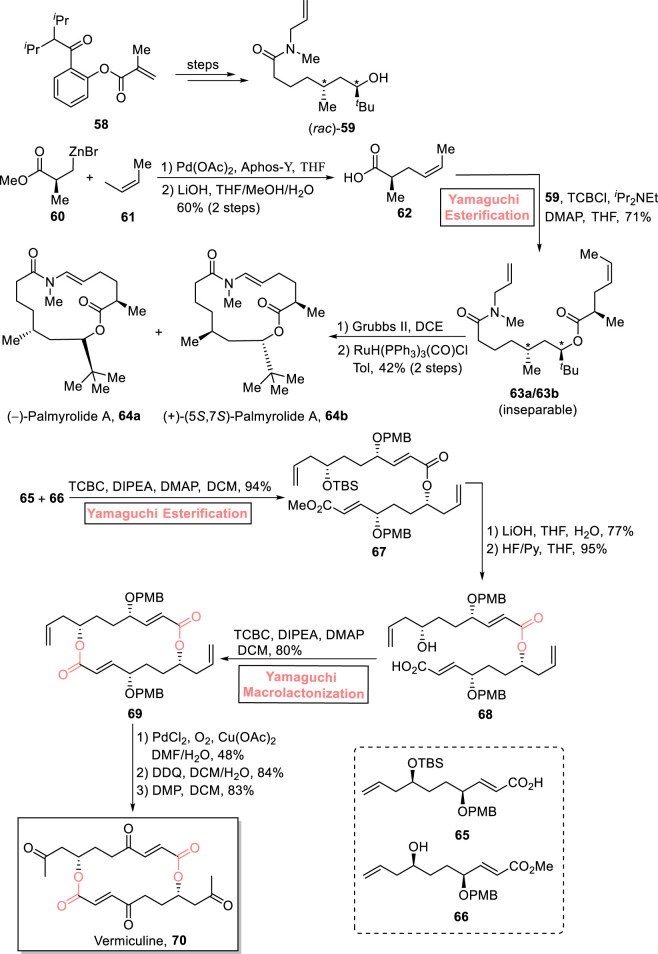
Synthesis of (−)-palmyrolide A **64a**, (+)-5,7-epi-palmyrolide A **64b**, and (−)-vermiculine **70**.

One of the neuroactive 15-membered macrolides, palmyrolide A, was isolated from an assembly of marine cyanobacteria consisting of *Oscillatoria* spp. and *Leptolyngbya* cf*.* It exhibits Ca influx suppression in cerebrocortical neurons (with an IC value of 3.70 μm) and Na channel suppression (with an IC value of 5.2 μm) (Pereira et al., 2010; Tan, 2007). Based on these captivating aspects, Lai and Dai (2021) enclosed the total synthesis of (−)-palmyrolide A **64a** and (+)-5,7-epi-palmyrolide A **64b** (Scheme 7) (Lai and Dai, 2021). Their multi-module strategic route toward synthesizing diasteroisomeric macrolides used Yamaguchi esterification as the main step. First, compounds **60** and **61** were subjected to the Negishi coupling reaction (in the presence of Pd(OAc)_2_, Aphos-Y, and THF), followed by hydrolysis to provide acid **62** with 60% yield. In the next steps, acid **62** was esterified with alcohol **59** (from starting material **58**) using a well-suited Yamaguchi protocol (TBSCl, ^*i*^Pr_2_Net, DMAP, and THF) to obtain an inseparable mixture of esters **63a** and **63b** with a combined yield of 71%. Furthermore, the ring-closing metathesis of compounds **63a** and **63b** (in the presence of Grubb’s second-generation catalyst) and subsequent treatment with RuH(PPh_3_)_3_(CO)Cl successfully provided (−)-palmyrolide A **64a** and (+)-5,7-epi-palmyrolide A **64b** with a combined yield of 42%.

#### 2.1.5 Synthesis of 16-membered macrolides

##### 2.1.5.1 Liu’s total synthesis of vermiculine

(−)-Vermiculine **70** is a 16-membered macrodiolide and was first isolated from *Penicillium vermiculatum* in 1972 by Kuhr et al. (Fuska et al., 1972). It holds significant medicinal importance owing to its anti-cancer, anti-protozoal, and immunomodulatory biological effects (Fuska et al., 1972; Fuska et al., 1974; Horáková et al., 1976). Liu et al. (2021) devised a highly flexible and efficient synthetic route for the synthesis of (−)-vermiculine **70** (as well as its analogs) in 14 steps and 9% overall yield (Scheme 7) (Liu et al., 2021). The cornerstone of their synthetic scheme lies in the utilization of Yamaguchi esterification (for the efficient ligation of compounds **65** and **66**) and Yamaguchi macrolactonization (for cyclization toward the construction of the 16-membered macrolide) as key steps. In their synthetic path, compounds **65** and **66** were subjected to Yamaguchi esterification in the presence of TCBC, diisopropyleyhylamine (DIPEA), DMAP, and DCM to achieve compound **67** with 94% yield. Dimer **67** was subjected to hydrolysis and TBS group removal to yield acid **68** (in 95% yield), which was proceeded further for macrolactonization under the Yamaguchi conditions, resulting in macrodiolide **69** with 80% yield. The oxidation of macrodiolide **69** in the presence of PdCl_2_, O_2_, and Cu(OAc)_2_, followed by PMB group removal and its Dess–Martin oxidation, resulted in the desired vermiculine **70** with 83% yield.

##### 2.1.5.2 Schmidt’s total synthesis of berkeleylactone A

**SCHEME 8:**
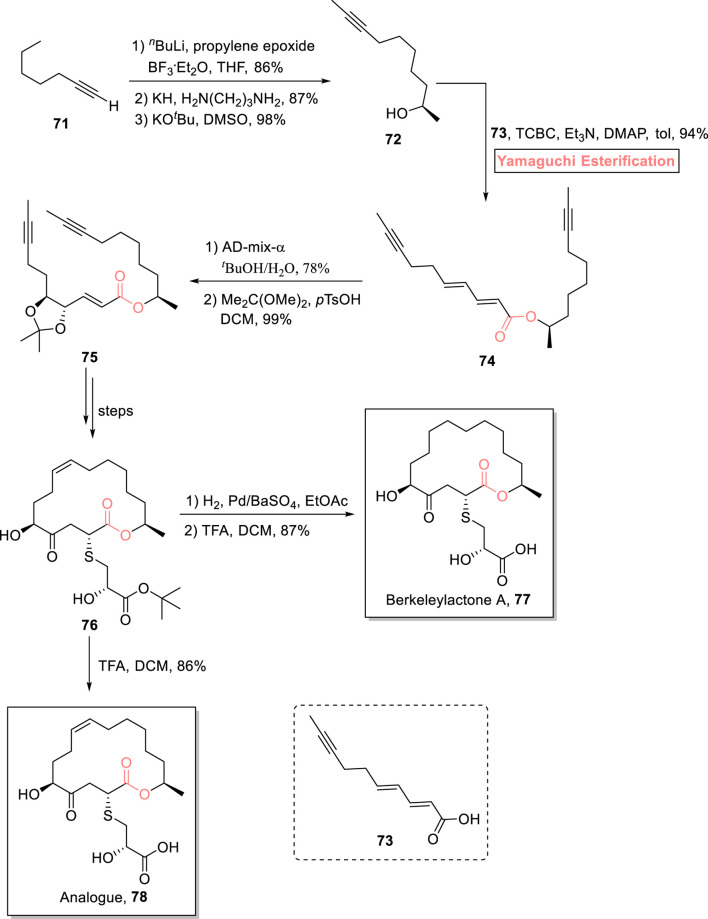
Synthesis of berkeleylactone A **77** and its analog **78**.

One of the 16-membered macrolides, berkeleylactone A **77**, was isolated from a fungal strain. It is a famous anti-biotic that exhibits a strong anti-microbial effect against multi-drug resistant *S. aureus* (Stierle et al., 2017; Michel et al., 1977; Vakiti et al., 2022). Schmidt et al. (2023) disclosed a novel synthetic route toward the synthesis of berkeleylactone A **77** and its analog **78** *via* easily accessible intermediate **76** (Scheme 8) (Schmidt et al., 2023). The whole methodology was started from alkyne **71**, which was reacted with propylene epoxide (under the given conditions) and then subjected to an alkyne zipper reaction, followed by treatment with potassium tertiary butoxide to result in alcohol **72** with 98% yield. In the next step, Yamaguchi esterification took precedence over Steglich esterification for furnishing ester in high yield as the substrate for te RCM reaction. Thus, the esterification of alcohol **72** with acid **73** was well managed with the exposure of the Yamaguchi reagent (TBSC, Et_3_N, and DMAP) in toluene as the solvent provided access to ester **74** with 94% yield. The dihydroxylation of compound **74** using AD-mix-α and subsequent acetonide protection resulted in compound **75** (in 99% yield), which was transformed into compound **76** over a few steps. Compound **76** was exposed to Rosenmund’s catalyst for hydrogenation and deprotection using TFA, resulting in berkeleylactone A **77** with 87% yield, while its analog was acquired by the direct deprotection of compound **76**. After the successful synthesis of both natural product **77** and its analog **78**, both of these were subjected to biological analysis against various bacterial and fungal strains, that is, *S. aureus*, *Candida glabrata*, *C. albicans*, *Enterococcus faecalis*, and *Enterococcus faecium*. Both compounds exhibited average-to-good anti-microbial effects against these strains.

##### 2.1.5.3 Kumari’s total synthesis of aspergillide D

**SCHEME 9:**
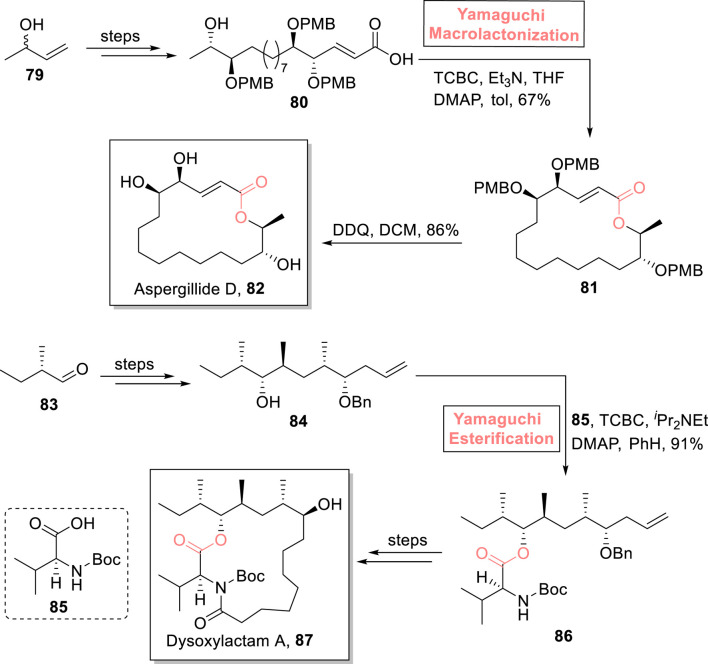
Synthesis of aspergillide D **82** and dysoxylactam A **87**.

Aspergillide D **82** is a 16-membered macrolide that was isolated from a fungal strain of *Aspergillus* sp. SCSGAF 0076. Its first total synthesis was performed by Mohapatra et al. in 2017 (Jena et al., 2017). After that, many reports on the total synthesis of this natural product have been published, with a common issue of low yield. Kumari et al. (2023) presented a high-yielding 15-step synthetic scheme for the synthesis of aspergillide D **82** *via* an epoxide ring-opening reaction (Ahmad et al., 2018) and Yamaguchi macrolactonization as the main steps (Scheme 9) (Kumari et al., 2023). In their synthesis, 3-butene-1-ol **79**, as a readily available compound, was utilized to produce hydroxy acid **80**. In order to perform macrolactonization, hydroxy acid **80** was treated with TCBC, Et_3_N, and DMAP in toluene to furnish highly regioselective lactone **81** with 67% yield. In the last step, deprotection was conducted in the presence of DDQ and DCM to produce the aspired aspergillide D **82** with 86% yield.

#### 2.1.6 Synthesis of 17-membered macrolides

##### 2.1.6.1 Yang’s total synthesis of dysoxylactam A

One of the 17-membered macrolide, dysoxylactam A **87**, was isolated from *Dysoxylum hongkongense*. It is a macrocyclic lipopeptide, consisting of a C19-branched fatty acid (Liu et al., 2019). Owing to its potential to reverse the P-glycoprotein-mediated multidrug resistance in tumor cells, Chandankar et al. performed its first total synthesis in 2020, and after that, various researchers have performed its total synthesis (Chandankar and Raghavan, 2020). In the continuation of these studies, Yang et al. (2022) designed an easy and concise synthetic route toward the total synthesis of this attractive unprecedented natural product (Scheme 9) (Yang et al., 2022). Their synthesis was based on 12 steps, starting from 2-methylbutanal **83**, and a 23.2% overall yield was acquired. After achieving compound **84** (from compound **83**), the Yamaguchi reagent (TCBC), ^*i*^Pr_2_NEt, and DMAP played their role in its esterification with compound **85** to successfully produce ester **86** (in 91% yield) with no epimerization. After several steps, dysoxylactam A **87** was easily obtained from ester **86**. The synthesized compound exhibited the cytotoxic activity in combination with vinorelbine (anti-cancer drug) with an IC_50_ value of 3.5 nmol. L^‒1^.

#### 2.1.7 Synthesis of 18-membered macrolactones

##### 2.1.7.1 Goda and Fuwa’s total synthesis of (−) enigmazole B

**SCHEME 10:**
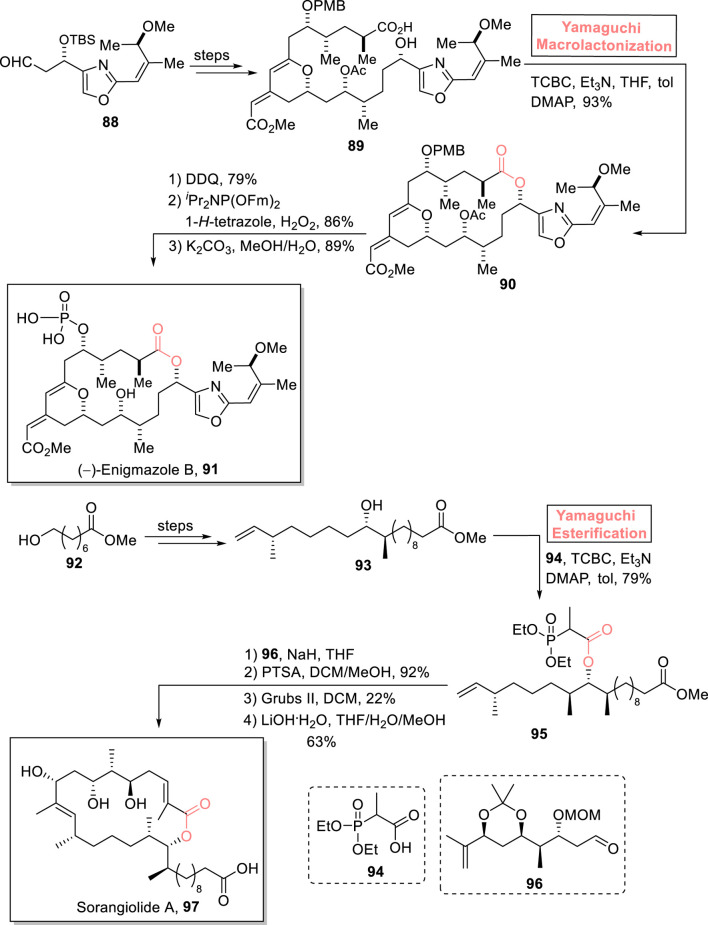
Synthesis of (−)-enigmazole B **91** and sorangiolide A **97**.

Among cytotoxic marine macrolides, enigmazoles were isolated from *Cinachyrella enigmatica* (Oku et al., 2010). (−)-Enigmazole A exhibits cytotoxic activity against a human cancer cell line, with GT_50_ = 1.7 μm. The intriguing anti-cancer biological profile of the enigmazole family gained the attention of many organic chemists (Takada et al., 2023). With profound interest, Goda and Fuwa, (2023) performed the first total synthesis of (−)-enigmazole B **91** in 20 consecutive steps using Yamaguchi macrolactonization as a key step (Scheme 10) (Goda and Fuwa, 2023). Their methodology was commenced with the modification of compound **88** over a few steps to provide compound **89**, which underwent macrocyclization *via* treatment with a well-suited Yamaguchi reagent in the presence of Et_3_N, THF, DMAP, and toluene, resulting in macrolactone **90** with 93% yield. Next, macrolactone **90** underwent a sequence of DDQ-mediated deprotection (of the PMB group), phosphorylation, and K_2_CO_3_-mediated deprotection to successfully yield the desired natural product (−)-enigmazole B **91** with 89% yield.

##### 2.1.7.2 Sahana’s total synthesis of sorangiolide A

Sorangiolide A **97** is an 18-membered macrolide and a polyketide that was isolated by Jansen et al. from *Sorangium cellulosum*, a myxobacterial strain. Structurally, the heterocyclic cage of sorangiolide A **97** consists of four methylated centers, two trisubstituted olefins, and four hydroxylated centers. This natural product is renowned for its anti-bacterial activity against *Staphylococcus aureus* (a Gram-positive bacterium with a MIC value of 5–10 μg/mL) (Jansen et al., 1995; Irschik et al., 1995). Sahana et al. (2022) designed a convergent asymmetric synthetic tool for the first successful total synthesis of sorangiolide A **97** (0.9% overall yield) by using Yamaguchi esterification as a key step (Scheme 10) (Sahana et al., 2023). In their synthetic route, alcohol **93** (prepared from compound **92**) was treated with triethyl amine, 2,4,6-trichlorobenzoyl chloride, DMAP, and toluene with the addition of acid **94**. This Yamaguchi protocol successfully produced ester **95** with 79% yield. Compound **95** was then made to couple with aldehyde **96** (in the presence of sodium hydride and THF), followed by acetonoid group deprotection (by using PTSA) to provide the intermediate with 92% yield. After that, it was subjected to RCM and subsequent treatment with LiOH^**.**^H_2_O to successfully produce the target product **97** with 63% yield.

##### 2.1.7.3 Salituro’s total synthesis of strasseriolides (A and B)

**SCHEME 11:**
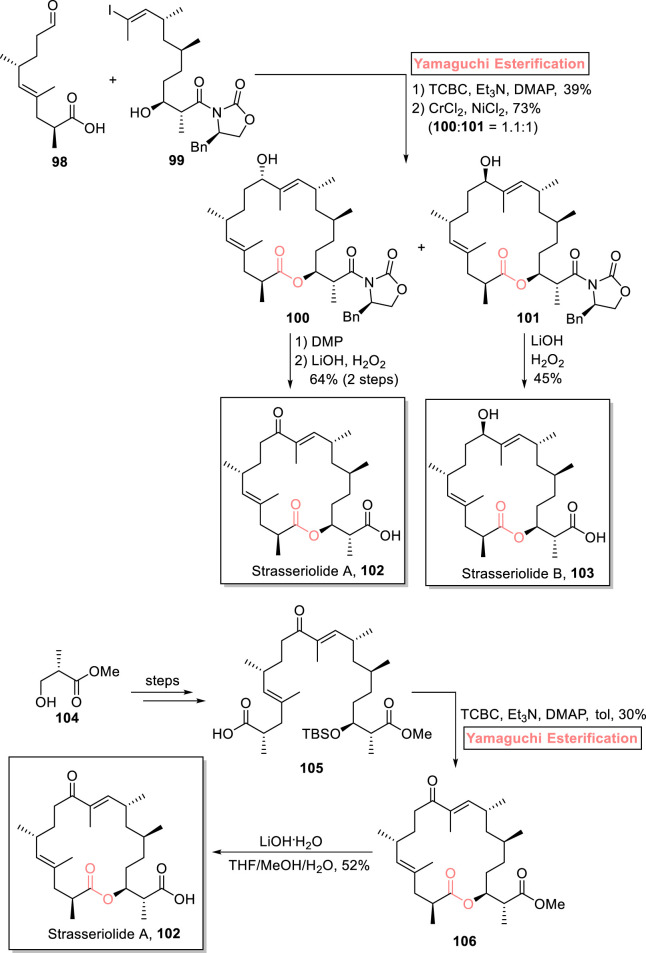
Synthesis of strasseriolide A **102** and strasseriolide B **103**.

Strasseriolides (A and B) were first isolated in 2020 by Rayes et al. from the fungal strain of *Strasseria geniculata* (CF-247251) in New Zealand (Annang et al., 2020). Structurally, they are 18-membered macrolides having 2 trisubstituted alkenes, 5 methyl centers, and 1 free carboxylic acid group (Saha et al., 2020). Strasseriolide B shows anti-malarial potential against *Plasmodium falciparum* (the most virulent parasite) (Pérez-Moreno et al., 2016). Inspired by this fact, Salituro et al. (2022) reported a robust synthetic route for the first total synthesis of strasseriolide A **102** and strasseriolide B **103** in 15-step and 16-step sequences, respectively (Scheme 11) (Salituro et al., 2022). The methodology entails Yamaguchi esterification and the Nozaki–Hiyama–Kishi (NHK) reaction as key steps commencing from readily available starting materials, i.e., acid **98** and alcohol **99**; both were explored for esterification under various protocols (EDCI, DCC, HBTU, and Shiina), but only the Yamaguchi method (TCBC, triethyl amine, and DMAP) was successful in the synthesis of the desired ester with 39% yield. The coupling intermediate underwent the NHK reaction to furnish compounds **100** and **101** with 73% combined yield (dr = 1.1:1). Compound **101** was oxidized in the presence of DMP, followed by hydrolysis, to produce strasseriolide A **102** with 64% yield. Furthermore, strasseriolide B **103** was easily acquired *via* the direct hydrolysis of compound **101**, with 45% yield.

##### 2.1.7.4 Sahana’s total synthesis of strasseriolide A

Pioneering the synthetic work on strasseriolides, Sahana et al. (2022) performed the total synthesis of strasseriolide A **102** in 22 steps with 1.0% overall yield (Scheme 11) (Sahana et al., 2022). In their synthesis, *S*-Roche ester **104** was used as the starting material, and Yamaguchi macrolactonization was used as a key step. After achieving compound **105** (from ester **104**), it was allowed to undergo macrolactonization following the Yamaguchi protocol (as the leading tool) by using TCBC, Et_3_N, DMAP, and toluene to obtain compound **106** (in 30% yield) with the desired stereochemistry. The whole synthetic scheme ended with the successful synthesis of strasseriolide A **102** (in 52% yield) by treating compound **106** with LiOH**·**H_2_O.

#### 2.1.8 Synthesis of 19-membered macrolides

##### 2.1.8.1 Zhang’s total synthesis of 27-deoxylyngbyabellin A

**SCHEME 12:**
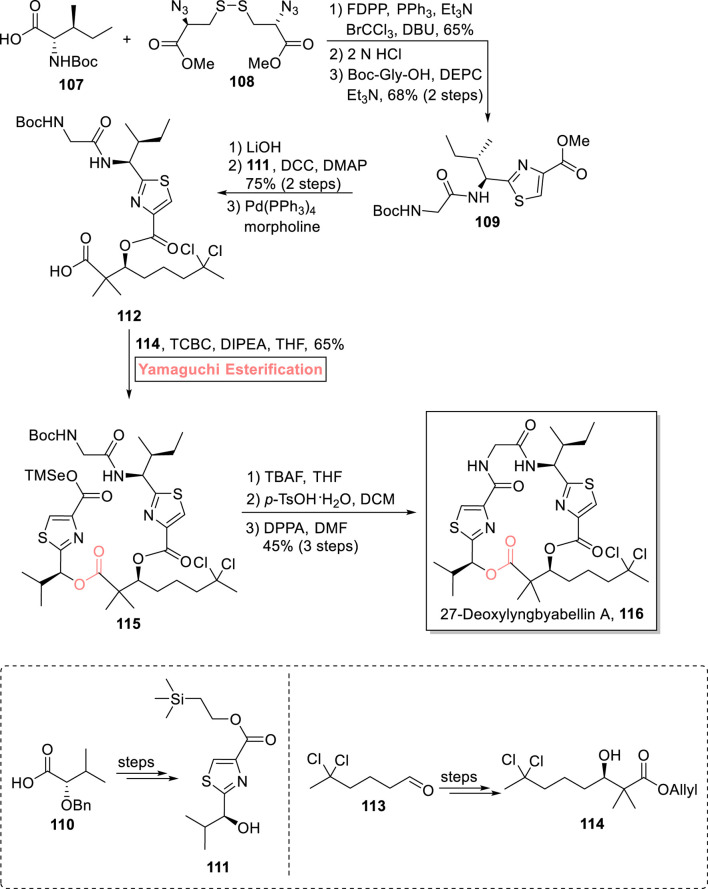
Synthesis of 27-deoxylyngbyabellin A **116**.

Lyngbyabellins are two thiazole rings containing marine metabolites, which are famous for their anti-cancer pharmaceutical effects (Pirovani et al., 2015). A 19-membered macrolide, 27-deoxylyngbyabellin A **116** was first isolated from *Lyngbya bouillonii* (a marine cyanobacterium) (Matthew et al., 2010). It exhibits cytotoxic effects against HeLa cervical cancer cells and HT-29 colorectal adeno cancer cells with IC_50_ values of 12 nM and 7.3 nM, respectively. Zhang et al. (2021) accomplished the total synthesis of 27-deoxylyngbyabellin A **116** in 10 consecutive steps with 9.7% overall yield (Scheme 12) (Zhang et al., 2021). Their efficient synthetic scheme began with the starting compounds **107** and **108**, which were allowed to react in the presence of pentafluorophenyl diphenylphosphinate, PPh_3_, and triethyl amine, followed by HCl-mediated Boc group removal and coupling with Boc-Gly-OH, which resulted in compound **109** with 68% yield. In the next step, compound **109** (after hydrolysis) was coupled with compound **111** (using DCC and DMAP), and the subsequent removal of allyl ester in the presence of morpholine and Pd(PPh_3_)_4_ produced acid **112**. In the next step of esterification of acid **112** with alcohol **114**, Keck esterification was attempted, but after its failure after a few attempts, the well-suited Yamaguchi reagent (2,4,6-trichlorobenzoyl chloride) was used in the presence of diisopropylethylamine as the base and THF as a solvent to result in the successful synthesis of ester **115** with 65% yield. After that, the TMSe and Boc groups were removed by treating ester **115** with TBAF and *p*-TsOH in a sequence, followed by its macrocyclization in the presence of diphenyl phosphorazidate and DMF to result in the successful synthesis of 27-deoxylyngbyabellin A **116** with 45% yield.

#### 2.1.9 Synthesis of 20-membered macrolides

##### 2.1.9.1 Gosh’s total synthesis of iriomoteolide-1a and iriomoteolide-1b

**SCHEME 13:**
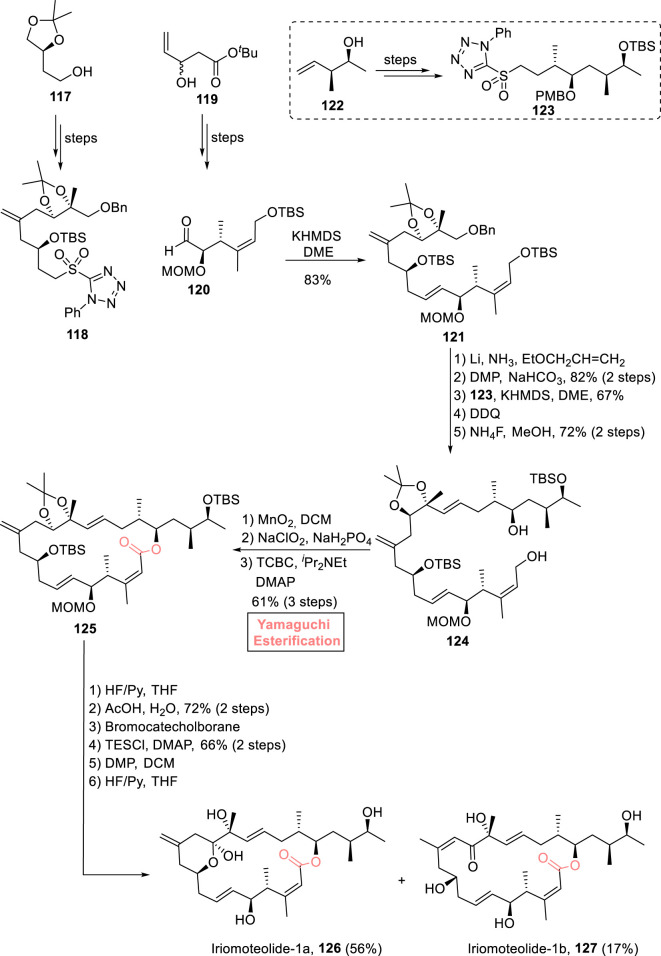
Synthesis of iriomoteolide-1a **126** and iriomoteolide-1b **127**.

Iriomoteolide-1a **126** and iriomoteolide-1b **127** are 20-membered macrolides that were isolated independently from the HY A024 strain of *Amphidinium* sp. (a dinoflagellate found in Japan) by Tsuda et al. (2007a) and Tsuda et al. (2007b). Both these natural marine products are structurally related to each other and are famous for their intriguing biological potential. In particular, iriomoteolide-1a exhibits cytotoxic activity against various human cell lines, that is, lymphocyte DG-75 and EBC-infected lymphocyte Raji cells with IC_50_ values of 2 ng/mL and 3 ng/mL, respectively (Munir et al., 2023). Considering these interesting facts, Gosh et al. (2022) devised a robust synthetic route for the synthesis of (the proposed structures) these natural products by using Yamaguchi macrolactonization as the key step (Scheme 13) (Ghosh and Yuan, 2022). Their synthesis was initiated with the synthesis of key fragments **118** and **120** (from starting materials **117** and **119**), followed by their coupling *via* the Julia–Kocienski reaction to result in olefin **121** with 83% yield. Olefin **121** (after benzyl group deprotection) was oxidized in the presence of DMP and NaHCO_3_. As a result, the oxidized product was then coupled with sulfone **123** (from alcohol **122**) *via* the Julia–Kocienski reaction, followed by PMB group and TBS group removal in sequence using DDQ and NaClO_2_, yielding alcohol **124** with 72% yield. Next, the oxidation of alcohol **124** first with MnO_2_ and second with NaClO_2_ adjusted the stage for Yamaguchi macrolactonization as the resulting carboxylic acid was treated with TCBC, DIPEA, and DMAP to successfully furnish 20-membered macrolactone **125** with 61% yield. The next few steps involved the replacement of the TBS group with the TES group, bromocatecholborane-induced deprotection of the MOM group, TES-protected oxidation of the free hydroxyl group (by using DMP), and a final deprotection in the presence of HF/Py, which resulted in the desired natural products iriomoteolide-1a **126** (in 56% yield) and iriomoteolide-1b **127** (in 17% yield).

##### 2.1.9.2 Bold’s synthesis of zampanolide analogs

**SCHEME 14:**
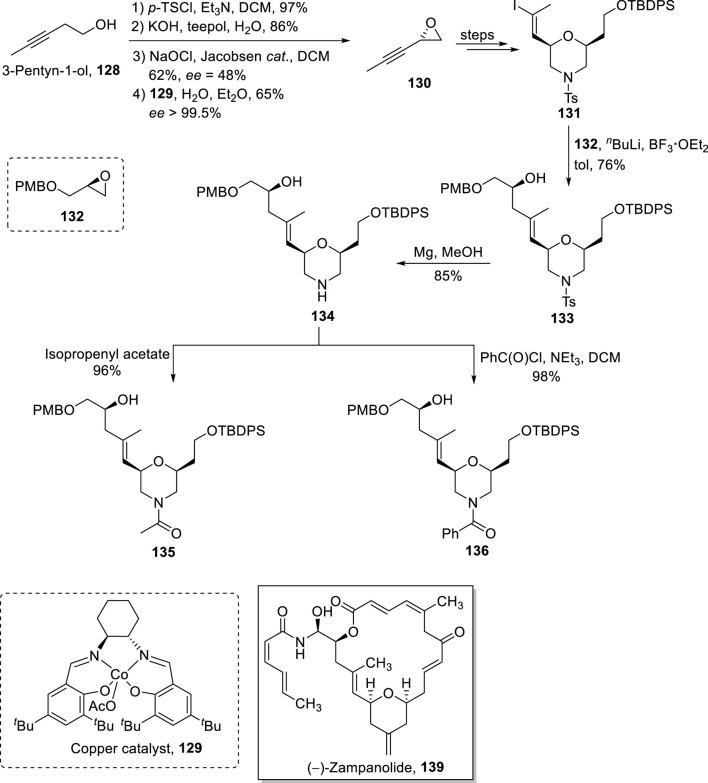
Synthesis of compounds **135** and **136** toward the synthesis of (−)-zampanolide analogs.

**SCHEME 15:**
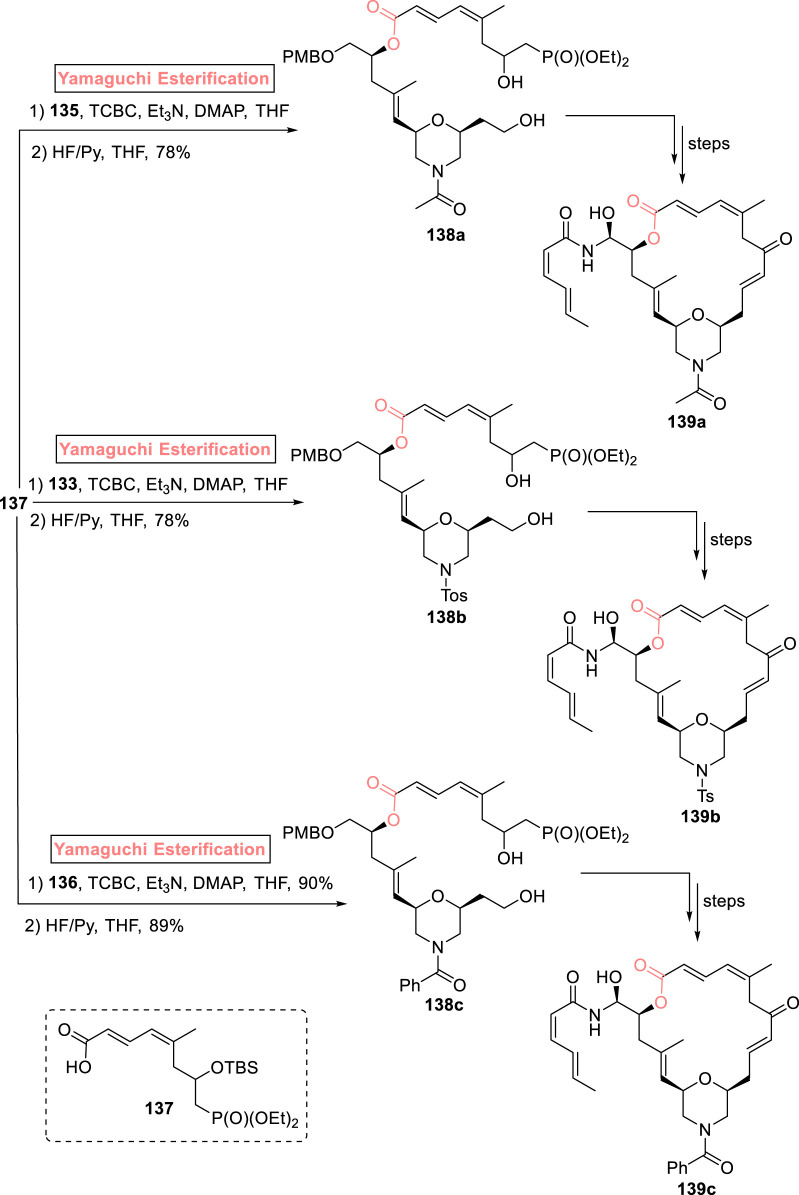
Synthesis of (−)-zampanolide analogs **139a–c**.

The famous anti-cancer (−)-zampanolide **139** is a 20-membered macrolide, which was isolated from *Fasciospongia rimosa* by Tanaka and Higa (1996). This natural product was re-isolated by Field et al. from *F. rimosa* in 1996 (Field et al., 2009). The structural activity studies of this unique pharmacophore underscore the necessity of developing analogs to maintain a broad-spectrum medicinal library (Chen and Kingston, 2014). In 2021, Bold et al. synthesized morpholine analogs of (−)-zampanolide with the desired stereochemistry that was assured *via* the Yamaguchi reagent (Scheme 15) (Bold et al., 2021). The synthetic scheme was initiated from alcohol **128** that was subjected to tosylation and treatment with a base (KOH), followed by Jacobsen epoxidation and copper catalyst-induced hydrolysis to yield epoxide **130** with 65% yield and 99.5% *ee*. After the transformation of epoxide **130** into alcohol **131**, it was reacted with epoxide **132** in the presence of butyl lithium and BF_3_ **·**OEt_2_, providing compound **133** in 76% yield. In the next step, the tosyl group of compound **133** was removed *via* treatment with magnesium, which led to the formation of compound **134** with 85% yield. Compound **135** (with 96% yield) was achieved from the acylation of compound **134** in the presence of isopropenyl acetate. Meanwhile, the reaction of compound **134** with benzoyl chloride in the presence of triethyl amine and DCM provided benzamide **136** with 98% yield (Scheme 14). The Yamaguchi esterification of the synthesized compounds **135**, **133**, and **136** with alcohol **137** (in the presence of TCBC, TEA, DMAP, and THF) and the subsequent deprotection step resulted in the independent synthesis of compounds **138a–c** with 78%–89% yields, which, after modification in some required steps, completed the synthesis of analogs **139a–c**.

##### 2.1.9.3 Umana’s synthesis of (−)-zampanolide analog

**SCHEME 16:**
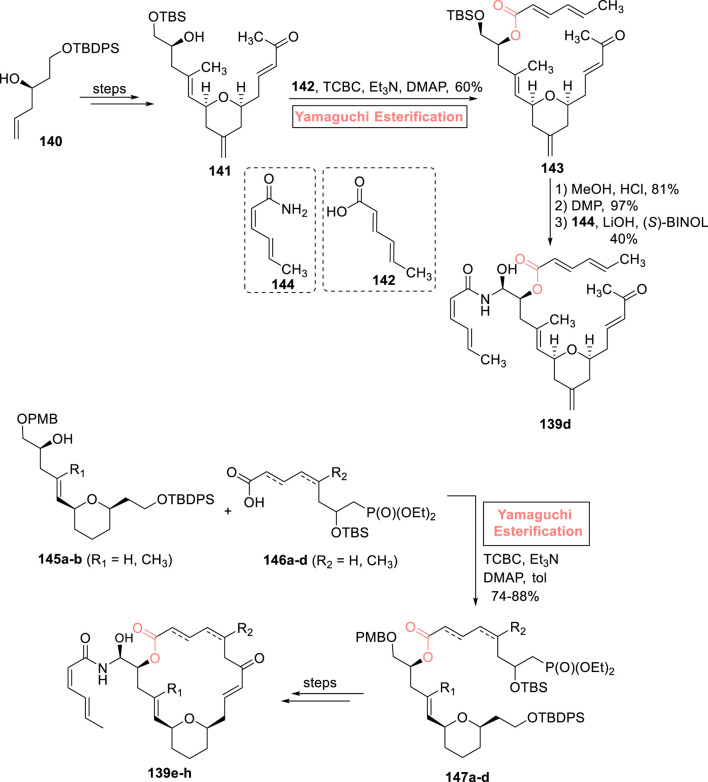
Synthesis of (−)-zampanolide analog **139d–h**.

In the continuation of work on (−)-zampanolide **139** analogs, Umana et al. (2023) developed a linear analog **139d** of (−)-zampanolide as a potential anti-cancer agent (Scheme 16). The synthesis was commenced from alcohol **140**, which was converted into compound **141** over a few steps. To set the stage for the incorporation of the side chain hemiaminal group, a challenging task with concern to attain the required configuration in the desired product, Yamaguchi esterification was preferred over any other step. Thus, alcohol **141** was made to undergo Yamaguchi esterification (with acid **142**) by treating it with 2,4,6-trichlorobenzoyl chloride, triethyl amine, and DMAP to achieve ester **143** with 60% yield. Compound **143** was treated with methanol and HCl (for silyl group deprotection), followed by DMP-induced oxidation and installation of a hemiaminal side group (*via* reaction with compound **144**), successfully producing the desired compound **139d** with 40% yield.

##### 2.1.9.4 Brutsch’s synthesis of (−)-zampanolide analogs

With the profound interest in the field of natural product synthesis, Brütsch et al. (2023) contributed their efforts to the synthesis of four desmethylene analogs of (−)-zampanolide **139d–h** (Scheme 16). Among these, the synthesis of three analogs involves the use of the efficient Yamaguchi reagent. The compounds **145a–b** were made to couple with compounds **146a–d** (macrocyclization) under the presented Yamaguchi conditions (TCBC, Et_3_N, DMAP, and toluene) to achieve the compounds **147a–d** within the yield range of 74%–88%. Over a few steps, (−)-zampanolide analogs **139e–h** were successfully attained from compounds **147a–d** (Supplementary Figure 3).

##### 2.1.9.5 Wender’s synthesis of bryostatin analogs

**SCHEME 17:**
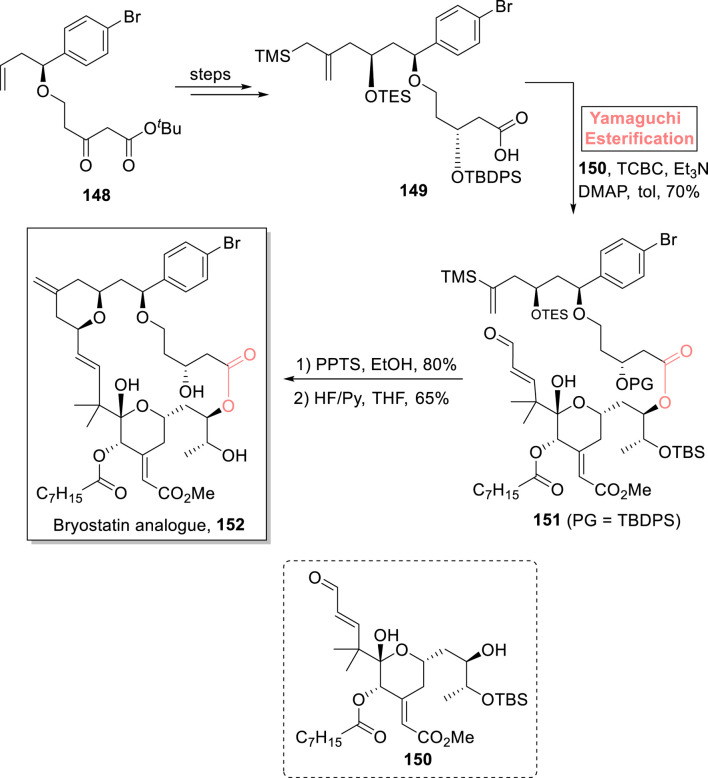
Synthesis of bryostatin analog **152**.

Bryostatin is a 20-membered polyketidic macrolide, which was isolated from *Bugula neritina*. It has various medicinal applications as it is used in the treatment of cancer, AIDS, Alzheimer’s disease, and many other degenerative diseases (Farlow et al., 2019; Gutiérrez et al., 2016). Considering the remarkable pharmacophore of bryostatin, Wender et al. (2022) introduced a novel strategy for the synthesis of its analog **152** (Scheme 17) (Wender et al., 2022). In their methodology, compound **149** (prepared from compound **148** in a few steps) esterified with alcohol **150** in the presence of a Yamaguchi reagent, Et_3_N, DMAP, and toluene, to obtain ester **151** with 70% yield. The deprotection of ester **151** in sequential steps using PPTS and HF/Py successfully produced bryostatin analog **152** with 65% yield.

#### 2.1.10 Synthesis of 22-membered natural macrolides

##### 2.1.10.1 Fritz’s total synthesis of pulvomycin D

A famous antibiotic, pulvomycin D, is a 22-membered macrolide that was isolated from *Streptomyces* sp. by Zief et al. (1957), and its structure was confirmed by Moon et al. (2020). This polyketidic macrolide also shows remarkable anti-cancer effects and has structural stability even under strong acidic and basic conditions (Kim et al., 1990). These interesting facts prompted Fritz et al. to perform its total synthesis in 2021 (Fritz et al., 2022). The synthesis of the precursor was achieved in consecutive steps with 0.23% overall yield *via* the use of a Yamaguchi reagent for the installation of the C1–C7 fragments (Supplementary Scheme 1).

#### 2.1.11 Synthesis of 23-membered natural macrolides

##### 2.1.11.1 Decultot and Clark’s total synthesis toward amphidinolide F

**SCHEME 18:**
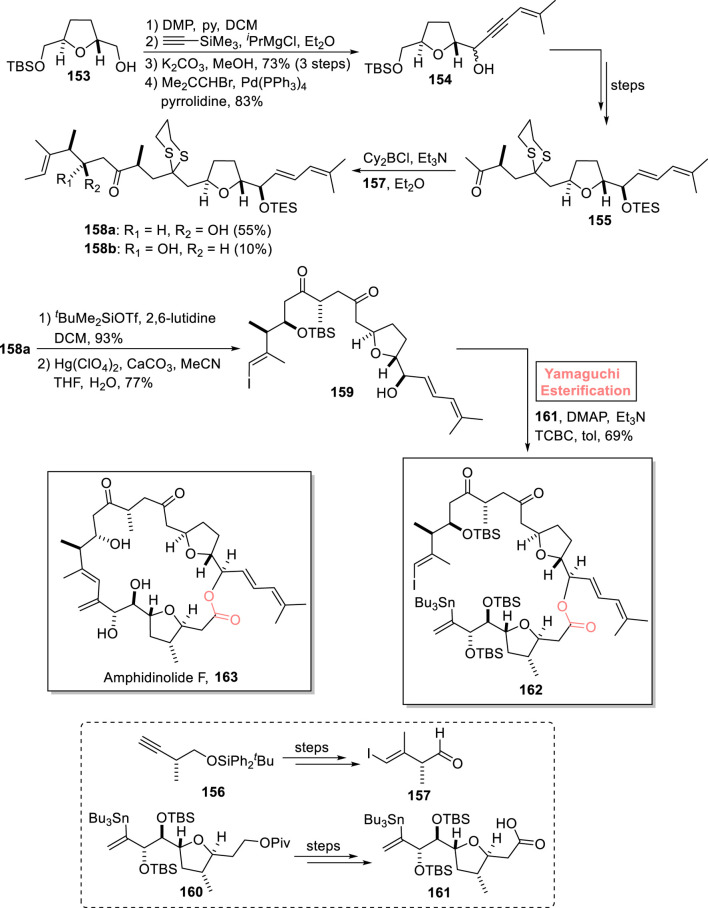
Synthesis of fragment **162** toward the synthesis of amphidinolide F **163**.

In 2022, Decultot and Clark performed the facile synthesis of precursor **162** toward the total synthesis of amphidinolide F **163** using Yamaguchi esterification as a key step (Scheme 18) (Decultot and Clark, 2022). In their synthetic path, alcohol **153** was subjected to DMP-promoted oxidation, followed by a reaction with the Grignard reagent, TMS group deprotection, and Sonogashira coupling reaction (with Me_2_CCHBr) to afford compound **154** with 83% yield. After a few steps, ketone **155** was acquired from compound **154**. Ketone **155** was allowed to react with aldehyde **157** in the presence of dicyclohexylboron chloride to generate a diastereomeric pair of alcohols **158a** and **158b** with 55% and 10% yields, respectively. In the next step, compound **158a** proceeded for TBS protection and subsequent hydrolysis, producing alcohol **159** with 77% yield. For the esterification of compound **159** with alcohol **161**, the well-optimized Yamaguchi protocol (Et_3_N, DMAP, and TCBC) was used to successfully produce fragment **162** with 69% yield.

#### 2.1.12 Synthesis of 28-membered polyketidic macrolides

##### 2.1.12.1 Babczyk and Menche’s total synthesis of pentamycin

**SCHEME 19:**
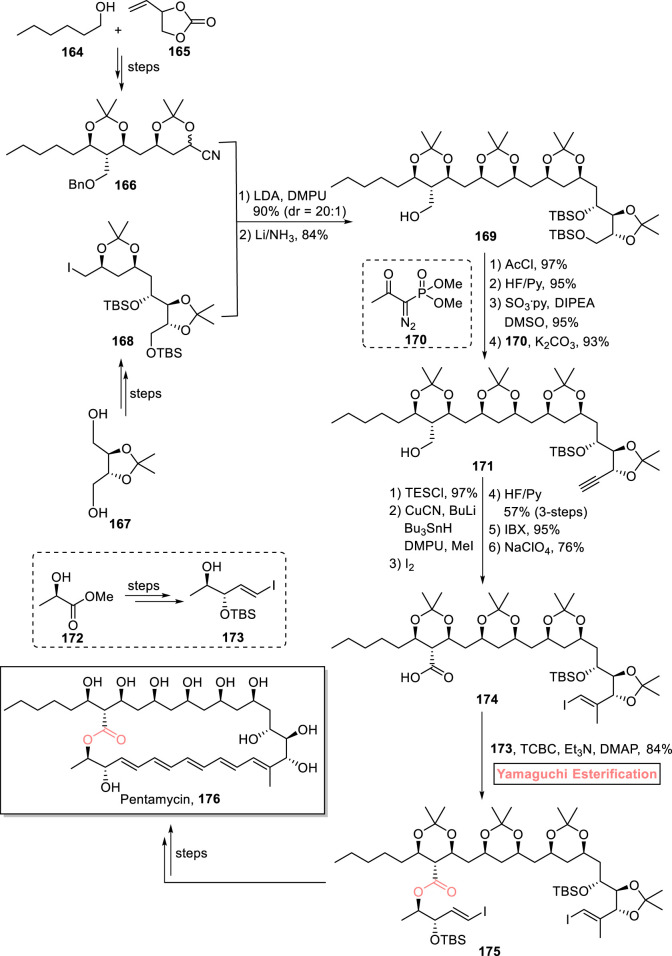
Synthesis of pentamycin **176**.

Pentamycin **176** belongs to the class of polyene macrolides and polyketides. It was first isolated in 1958 from *Streptomyces pentaticus* (Hamilton-Miller, 1973). The unique structural framework of this macrolide consists of a 28-membered core with an adjacent polyol fragment, having 12 stereomeric centers. Pentamycin **176** exhibits remarkable biological activities against *C. albicans* and *Trichomonas vaginalis*. Furthermore, it can also be used as an anti-cancer agent along with bleomycin (Kranzler et al., 2015; Payero et al., 2015). These fascinating features prompted Babczyk and Menche to perform its total synthesis by using Yamaguchi esterification as the crucial step (Scheme 19) (Babczyk and Menche, 2023). For the accomplishment of this task, fragment **166** (from compounds **164** and **165**) and fragment **168** (from compound **167**) were subjected to coupling reaction (in the presence of LDA and DMPU), followed by Birch reduction to produce compound **169** in 84% yield. In the four steps for the synthesis of alkyne **171**, acetyl group protection and TBS group deprotection of compound **169** provided favorable conditions for subsequent oxidation and Bestmann–Ohira homologation (with compound **170**). Alkyne **171** was then subjected to a sequence of stannyl-cupration (under TES protection), methylation, iodination, IBX-induced oxidation, and Pinnick oxidation to furnish compound **174** with 76% yield. To perform the challenging esterification of this sterically hindered carboxylic acid **174** with alcohol **173**, the well-suited Yamaguchi protocol was used by using TCBC, Et_3_N, and DMAP, which yielded ester **175** with 84% yield. After a few steps, the successful synthesis of the targeted pentamycin **176** was achieved gratifyingly.

#### 2.1.13 Synthesis of 30-membered macrolides

##### 2.1.13.1 Lizzadro’s synthesis of disorazole C_1_ and analogs

**SCHEME 20:**
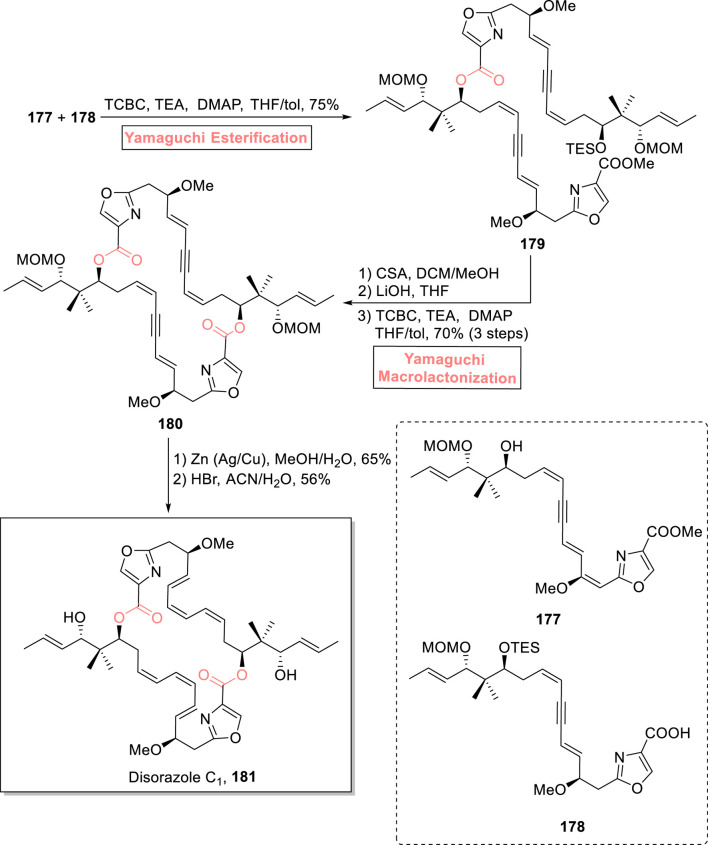
Synthesis of disorazole C_1_ **181**.

Disorazoles are an intriguing class of natural products well known for their anti-tubulin and anti-cancer effects. They were isolated in 1994 from *S. cellulosum*, a myxobacterium, by Hofle and Reichenbach (Jansen et al., 1994; Jensen, 1994). One of the disorazoles, (−)-disorazole C_1_ **181**, is a 30-membered macrolide. It exhibits a highly cytotoxic effect in various mammalian cell lines (Hopkins and Wipf, 2009). Lizzadro et al. (2021) devised an efficient and well-designed strategy for the total synthesis of (−)-disorazole C_1_ (Scheme 20) (Lizzadro et al., 2021). The interesting features of their synthetic scheme involved both Yamaguchi esterification and Yamaguchi macrolactonization as the main steps to obtain the 30-membered macrolide (Supplementary Scheme 2). The Yamaguchi esterification was made possible by reacting synthesized compounds **177** and **178** in the presence of 2,4,6-trichlorobenzoyl chloride, triethyl amine, DMAP, and toluene, producing ester **179** with 75% yield. For the intramolecular reaction, a sequence of silyl group deprotection and hydrolysis of ester **179**, followed by exposure to Yamaguchi conditions for macrolactonization, resulted in cyclic macrocycle **180** with 70% yield. In the end, metal-catalyzed reduction of compound **180**, followed by a reaction with HBr in acetonitrile and water, resulted in the synthesis of the targeted natural product **181** with 56% yield. After the successful synthesis of (−)-disorazole C_1_ **181**, Lizzadro et al*.* (2022), extended their methodology for the synthesis of three novel analogs of disorazole as potent cytotoxic agents against cancer (Lizzadro et al., 2022) (Supplementary Figure 4).

### 2.2 Synthesis of natural metabolites

#### 2.2.1 Gillsch’s total synthesis of ophiofuranones (A and B)

Ophiofuranone A and ophiofuranone B, belonging to the class of fungal metabolites, were isolated from *Ophiosphaerella korrae* by Lou et al. (2019) (Li et al., 2019). In 2022, Gillsch et al*.* disclosed the first total synthesis and microbial analysis of ophiofuranone A and ophiofuranone B by using cheap starting materials, i.e., tiglic acid and methallyl alcohol (Gillsch et al., 2022b). The challenging molecular architecture (with 4 stereogenic centers) of these 2 natural products was easily built in 16 steps by using Yamaguchi esterification as a powerful step (Supplementary Scheme 3).

#### 2.2.2 Gillsch’s total synthesis of thiocarboxylic acid and analogs

A fungal metabolite, thiocarboxylic acid, was isolated from *Penicillium* sp. Sb62. This unique natural product exhibits anti-microbial activity against *S. aureus*, *Escherichia coli*, and *C. albicans* with MIC values of 1.7–3.0 μg/mL (Chang et al., 2020). Gillsch et al. (2022) devised an efficient 14-step synthetic route toward the synthesis of thiocarboxylic acid and its three analogs (Gillsch et al., 2022a). The key step entails the Yamaguchi esterification, which assisted in the separation of *E* and *Z* isomers with no scrambling (Supplementary Scheme 3).

#### 2.2.3 Wittman’s synthesis of the JBIR-141 analog

**SCHEME 21:**
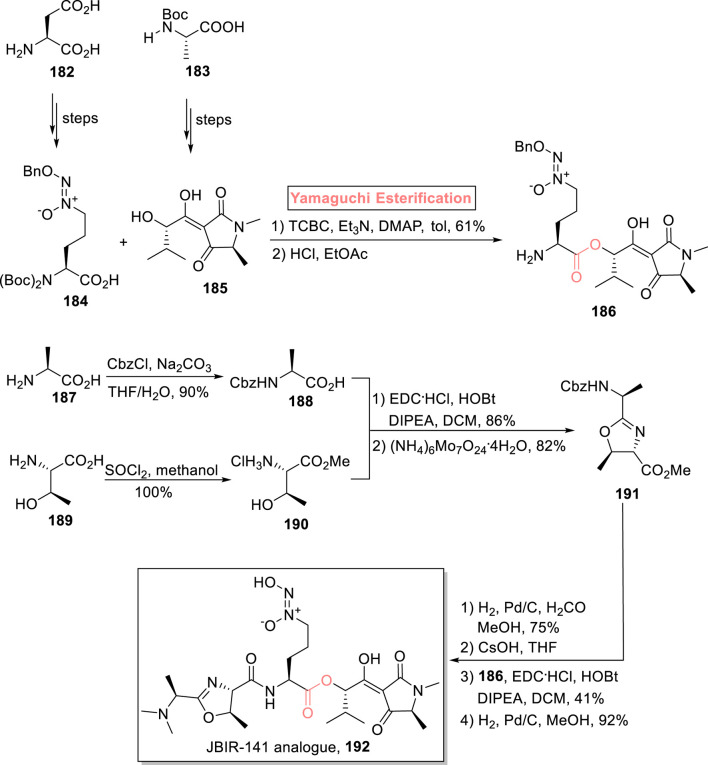
Synthesis of JBIR-141 **192**.

Cancer is the second leading cause of death, and it has numerous causes. So, the search for new anti-cancer drugs with improved cytotoxicity has always remained a significant interest of scientists. Among some anti-cancer compounds, JBIR-141 inhibits the transcription of Foxo3a with an IC_50_ value of 23.1 nM. It is a tetramic acid metabolite that was isolated from the 4587H4S strain of *Streptomyces* sp. Furthermore, this natural product possesses significant structural features for having *N*-nitrosohydroxylamine, 3-acyltetramic acid, and oxazoline-4-carboxamide adorned with six stereogenic centers (Kawahara et al., 2015; Yasoshima et al., 2022). With great interest, Wittman et al. (2022) devised an efficient strategy for the synthesis of a close analog of JBIR-141245 using readily available starting materials _L_-threonine **189**, _L_-alanine **187**, and _L_-glutamic acid **182** (Scheme 21) (Wittmann et al., 2022). The synthesized compounds **184** and **185** (from starting compounds **182** and **183**), in hand, were subjected to Yamaguchi conditions (TCBC, Et_3_N, DMAP, and toluene), facilitating a smooth esterification process. The resulting ester was then treated with HCl and ethyl acetate to yield compound **186**. For the synthesis of key fragment **188**, compound **187** was protected by using carbazole chloride. On the other hand, compound **189** was turned into ester **190** *via* its treatment with SO_2_ and methanol. The resulting ester (in 100% yield) was then made to react with protected compound **188** under the given conditions for condensation, followed by cyclization in the presence of (NH_4_)_6_Mo_7_O_24_^**.**^4H_2_O, to provide dipeptide **191** with 82% yield. Next, the removal of the carbazole group of compound **191** was made possible by hydrogenolysis in the presence of formaldehyde. This was followed by CsOH-promoted saponification, coupling of the intermediate with ester **186** (in the presence of EDC, DIPEA, HOBt, and DCM), and a final step of debenzylation, leading to the target analog **192** with 92% yield.

#### 2.2.4 Jansen’s total synthesis of desferri-exochelin 772SM

**SCHEME 22:**
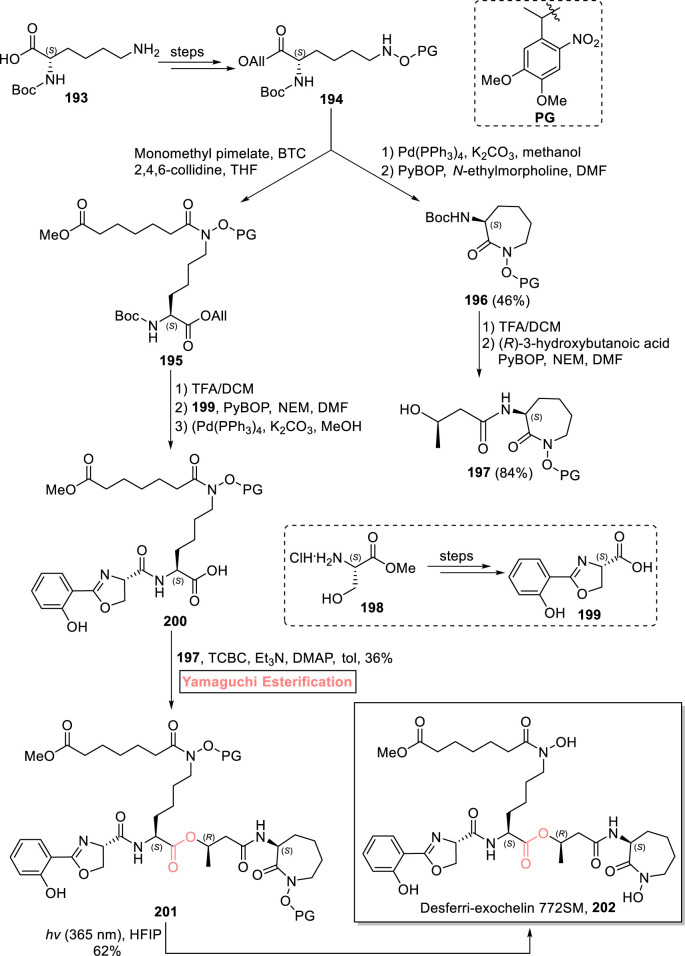
Synthesis of desferri-exochelin 772SM **202**.

Exochelins are mycobacterial secretions that are used to chelate iron (as is necessary for the replication and energy metabolism of these microorganisms). One of these natural products, desferri-exochelin 772SM **202**, was first isolated by Horwitz et al. from *M. tuberculosis* in 1995 (Horwitz and Horwitz, 2014; Gobin et al., 1995). Their significant Fe-chelating capability prompted Jansen et al. (2023) to devise a convergent and concise synthetic route for the total synthesis of desferri-exochelin 772SM **202** in 11 consecutive steps with an 8.6% overall yield (Scheme 22) (Jansen et al., 2023). The key step in the synthesis entails the coupling of advanced, hugely decorated fragments using Yamaguchi esterification. In their methodology, the easily available starting material **193** was transformed into compound **194** over a few steps. On one hand, compound **194** was used for the synthesis of alcohol **197** with the assistance of Pd(PPh_3_)_4_-mediated deprotection and PyBOP-induced coupling with *N*-ethylmorpholine of compound **194**, resulting in intermediate **196** (with 46% yield). Subsequently, the Boc group protection of compound **196** was performed, followed by its PyBOP-induced coupling with 3-hydroxybutanoic acid, resulting in cobactin **197** with 84% yield, while in another route, compound **194** was made to react with monomethyl pimelate bis-(trichloromethyl)carbonate, 2,4,6-collidine, and THF to yield compound **195**, proceeded by Boc-group deprotection, PyBOP-assisted amide coupling (with compound **199**), and allyl group deprotection, to provide acid **200** (ready to esterify). Next, the essence of the Yamaguchi protocol can be realized as it was applied for the esterification (after the failure of other methods) of compound **200** with already synthesized alcohol **197** in the presence of TCBC, Et_3_N, DMAP, and toluene to produce ester **201** in 36% yield. In the final step, ester **201** was treated with hexafluoroisopropanol (HFIP) in the presence of sunlight to successfully produce desferri-exochelin 772SM **202** with 62% yield.

### 2.3 Synthesis of natural polyketides

#### 2.3.1 Morishita’s total synthesis of nhatrangin A

**SCHEME 23:**
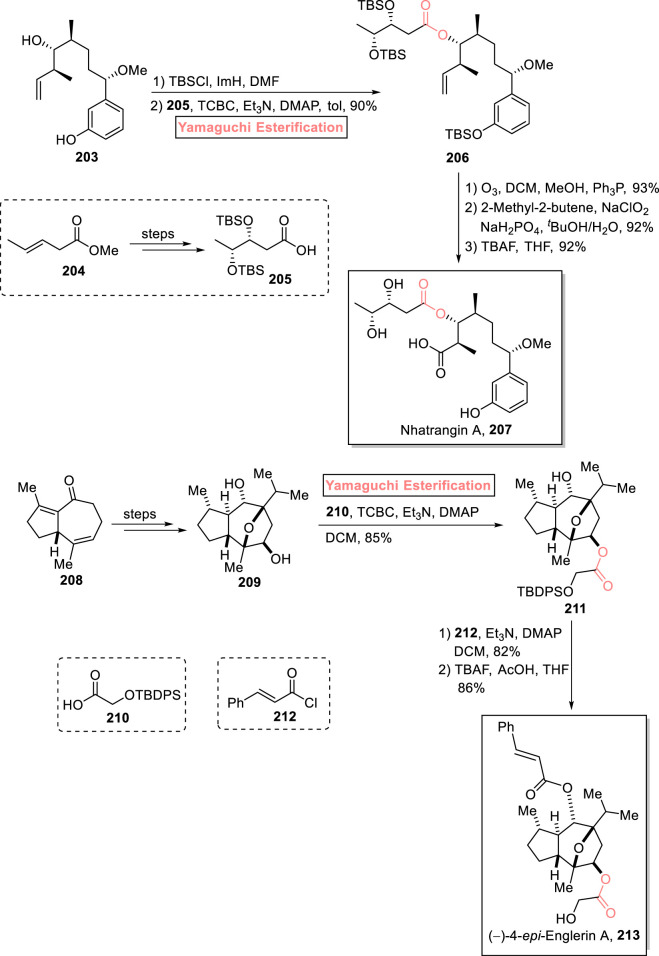
Synthesis of nhatrangin A **207** and (−)-4-epi-englerin A **213**.

Nhatrangin A **207** belongs to the class of polyketide natural products and is a truncated derivative and a synthetic intermediate of oscillatoxin D and aplysiatoxin. Nhatrangin A **207** was first isolated from *Lyngbya majuscula*, a marine cyanobacterium, by Orjala et al. in 2010 (Chlipala et al., 2010). More interestingly, aplysiatoxin is renowned for its anti-inflammatory and anti-tumor activities (Fujiki et al., 1984). Being the synthetic intermediate of aplysiatoxin, nhatrangin A **207** gained the attention of various research groups; Morishita et al. (2023) accomplished its total synthesis and confirmed its configuration with a previously reported synthesis (Scheme 23) (Morishita et al., 2023). Their synthesis commenced with the efficient Yamaguchi esterification of compound **203** (after silyl protection) with compound **205** (from compound **204**) in the presence of TCBC, Et_3_N, DMAP, and toluene to yield compound **206** with 90% yield. Subsequently, compound **206** was subjected to ozonolysis (in the presence of ozone and triphenyl phosphine), Pinnick oxidation (with 2-methyl-2-butene), and TBS-group deprotection in sequence to successfully produce the targeted nhatrangin A **207** in 92% yield.

### 2.4 Synthesis of natural terpenoids

#### 2.4.1 Kumar Palli’s total synthesis of (−)-4-epi-englerin A

The natural extracts of the plant *Phyllanthus engleri* are renowned for their cytotoxic activity against renal cancer cells (Ratnayake et al., 2009). One of these natural products includes (−)-4-epi-englerin A **213**, which is a sesquiterpenoid. The first total synthesis of this medicinally important scaffold was performed by Christmann in 2009 (Willot et al., 2010). Kumar Palli et al. (2021) also reported a valuable approach (based on 22 steps) for its total synthesis (with 4% overall yield) by using Yamaguchi esterification as the crucial step (Scheme 23) (Kumar Palli et al., 2021). Their synthesis started with compound **208**, which was modified into diol **209** through a few steps. Next, the standard conditions for strategic Yamaguchi esterification were adjusted by reacting diol **209** with protected alcohol **210** in the presence of TCBC, Et_3_N, DMAP, and CH_2_Cl_2_ (solvent) to attain ester **211** with 85% yield. Then, ester **211** was allowed to react with trans-cinnamoyl chloride **212,** followed by desilylation to gain the desired (−)-4-epi-englerin A **213** with 86% yield.

### 2.5 Synthesis of natural peptides

#### 2.5.1 Chen’s total synthesis of colletopeptide A and colletotrichamide A

**SCHEME 24:**
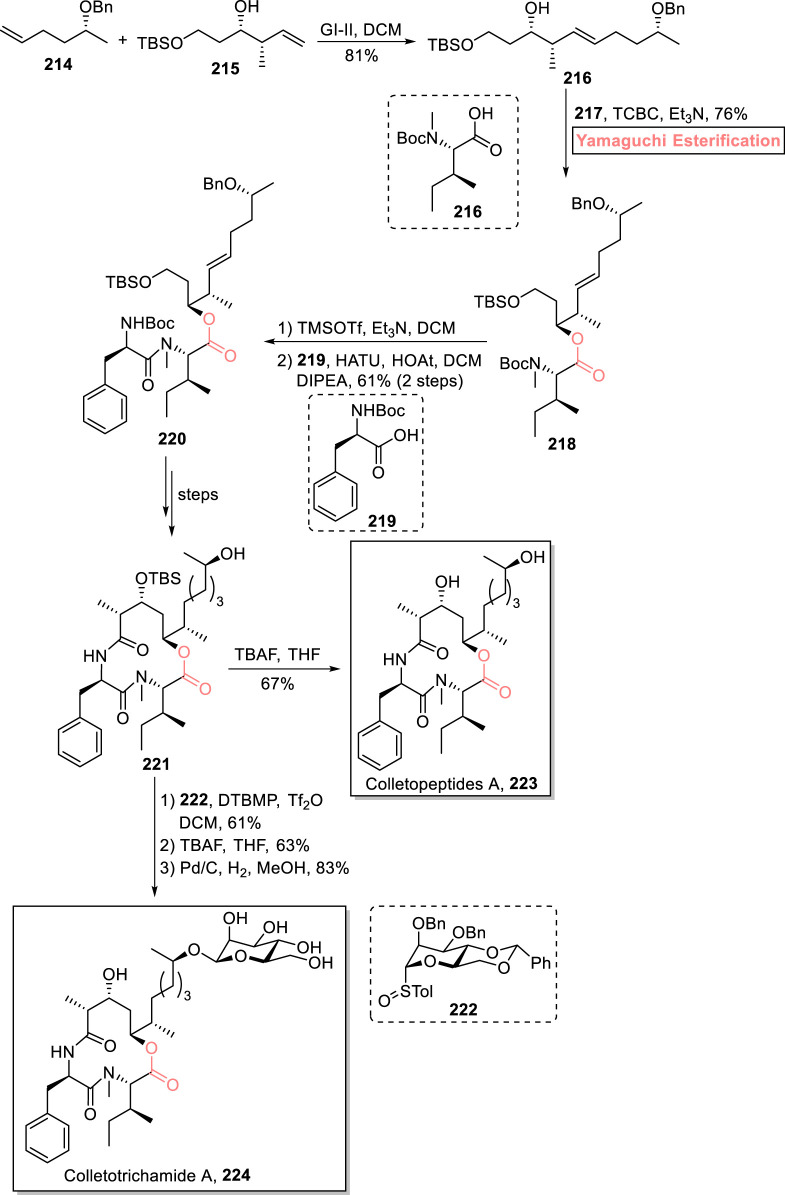
Synthesis of colletopeptide A **223** and colletotrichamide A **224**.

Colletopeptide A **223** and colletotrichamide A **224** are cyclic depsipeptides that were isolated independently from *Colletotrichum gloeosporioides* JS419 and *Colletotrichum* sp. S8 (Feng et al., 2019). These natural products are renowned for their broad range of biological activities. In particular, colletopeptide A **223** exhibits cytotoxic activity against RAW264.7 macrophages with IC_50_ = 8.3 μM (Oh et al., 2006; Ohno et al., 2004). With a profound interest in these medicinally active peptides, Chen et al. (2023) devised an impressive strategy for the first total synthesis of colletopeptide A **223** (in 15 steps) and colletotrichamide A **224** (in 17 steps) *via* a common precursor **221** (Scheme 24) (Chen et al., 2023). Their successful stereoselective synthesis entails Yamaguchi esterification as the main step. In the first step, the easily available starting materials, i.e., alkenes **214** and **215**, were subjected to cross-metathesis to produce alkene **216** with 81% yield. Next, ester **218** was easily obtained in the desired stereochemistry using the Yamaguchi protocol for esterification. For this, compounds **216** and **217** were treated with TCBC and Et_3_N, yielding ester **218** with 76% yield. Compound **218** was treated with TMSOTf (for removal of the Boc group), and its amide coupling reaction with compound **219** led to the synthesis of tridepsipeptide **220** with 61% yield. It took a few steps for the achievement of precursor **221**, which, after TBS removal, directly furnished colletopeptide A **223** with 67% yield. However, for the synthesis of colletotrichamide A **224**, the precursor **221** was allowed to react with mannose derivative **222** in the presence of DTBMP, Tf_2_O, and DCM, followed by sequential steps involving benzyl group and TBS group removal under given conditions, to successfully achieve the desired natural product **224** with 83% yield.

#### 2.5.2 Bauer and Kazmaier’s total synthesis of thiamyxins (A, B, C, and E)

Thiamyxins (A, B, E and C) are depsipeptides in nature, and these were isolated by Muller from a myxobacterial variant of the Myxococcaceae (MCy9487) family (Haack et al., 2022). These thiazoline-containing natural products are popular owing to their anti-viral activities (more particularly against Zika, dengue, and Coronavirus). These interesting features prompted Bauer and Kazmaier (2023) to perform their challenging total synthesis by using Yamaguchi esterification as a key step for macrolactonization with the careful embellishment of stereocenters (Supplementary Scheme 3) (Bauer and Kazmaier, 2023).

### 2.6 Synthesis of natural α-pyrone

#### 2.6.1 Zhao’s total synthesis of brevipolide H

Brevipolides A–J are α-pyrone-based natural products. Brevipolides G–I were isolated from *Lippia alba* by Hegde et al. (2004). Structurally, these are dihydro-α-pyrone motifs containing a cyclopropane ring in their side chain. This family of natural products shows anti-cancer activities against various cell lines (Deng et al., 2009). Considering their impressive pharmaceutical profile, Zhao et al. (2023) conducted the total synthesis of brevipolide H **293** in 12 consecutive steps with 8.65% overall yield (Zhao et al., 2023). The methodology was based on the synthesis of allylic alcohol **291** starting from _D_-galactal **289**, covering a few steps. Next, envisaging the Yamaguchi protocol as a leading tool for esterification, compound **291** was subjected to Et_3_N, TCBC, and DMAP with the addition of *p*-methoxycinnamic acid to provide ester **292** with 73% yield. In the final step, HCl-mediated acetyl group deprotection of ester **292** produced brevipolide H **293** with 77% yield.

### 2.7 Synthesis of natural biosurfactants

#### 2.7.1 Mittendorf’s total synthesis of (+)-aureosurfactin 1a and (−)-aureosurfactin 1b

Biosurfactants are important (ecofriendly) natural products, with extensive applications in the textile, cosmetic, food, and pharmaceutical industries (Singh et al., 2007; Mulligan, 2005). One of the biosurfactants, aureosurfactin, was isolated first by Yun et al. from *Aureobasidium pullulans* in 2016 (Kim et al., 2016). Structurally, aureosurfactin is an ester of acyclic dimer 3,5-dihydroxydecanoic acid. In 2023, Mittendorf *et al.* devised a concise strategy for the total synthesis of both enantiomers, i.e., (+)-aureosurfactin **299a** and (−)-aureosurfactin **299b** with 18% and 13.5% overall yields, respectively (Mittendorf et al., 2023). In their synthetic scheme, valeraldehydes **294a** and **294b** (as starting materials), in a few steps, provided acids **296a** and **296b** (independently), which further underwent methylation and TES group deprotection to result in alcohols **297a** and **297b** in 78% and 67% yields, respectively. Next, the key fragments **296a** and **297b,** as well as **296b** and **297b,** were subjected to coupling *via* the use of a well-compatible reagent, i.e., TBSCl, Et_3_N, and toluene (Yamaguchi esterification) to generate esters **298a** and **298b** with 92% and 93% yields, respectively. Finally, an additional HF/Py-mediated deprotection step (independently) generated both enantiomers, namely, (+)-aureosurfactin **299a** and (−)-aureosurfactin **299b**, in 99% and 79% yields, correspondingly.

## 3 Conclusion

The formal and total syntheses, structural elucidation, and revision of (newly isolated, as well as previously existing) natural products are meticulous steps in the design and development of drugs. In this endeavor, many organic reactions and reagents play prominent roles, as reported in the literature. The Yamaguchi reagent (2,4,6-trichlorobenzoyl chloride) is one among such reagents, and it is responsible for esterification between an acid and alcohol in the presence of DMAP as a coupling reagent. This reagent (despite its limitations including low yield and high cost) has been efficiently used in the synthesis of a wide variety of esters and lactones that are present in various biologically active natural products as key structural motifs. Our article presents an up-to-date compilation of Yamaguchi reagent-based synthetic schemes utilized for the synthesis of natural products and is aimed at helping related research groups in possibly devising further synthetic routes toward the synthesis of a diverse range of natural products (whose total syntheses are yet to be unlocked) using this reagent.

## References

[B1] Abid-Essefi S. Ouanes Z. Hassen W. Baudrimont I. Creppy E. Bacha H. (2004). Cytotoxicity, inhibition of DNA and protein syntheses and oxidative damage in cultured cells exposed to zearalenone. Toxicol. vitro 18 (4), 467–474. 10.1016/j.tiv.2003.12.011 15130604

[B2] Ahmad S. Zahoor A. F. Naqvi S. A. R. Akash M. (2018). Recent trends in ring opening of epoxides with sulfur nucleophiles. Mol. Divers. 22 (1), 191–205. 10.1007/s11030-017-9796-x 29138964

[B3] Allu S. R. Banne S. Jiang J. Qi N. Guo J. He Y. (2019). A unified synthetic approach to optically pure curvularin-type metabolites. J. Org. Chem. 84 (11), 7227–7237. 10.1021/acs.joc.9b00776 31083915

[B4] Annang F. Pérez-Moreno G. Gonzalez-Menendez V. Lacret R. Pérez-Victoria I. Martín J. (2020). Strasseriolides A–D, a family of antiplasmodial macrolides isolated from the fungus Strasseria geniculata CF-247251. Org. Lett. 22 (17), 6709–6713. 10.1021/acs.orglett.0c01665 32808790

[B5] Babczyk A. Menche D. (2023). Total synthesis of pentamycin by a conformationally biased double stille ring closure with a trienyl-bis-stannane. J. Am. Chem. Soc. 145 (20), 10974–10979. 10.1021/jacs.3c03011 37162233

[B6] Bai Y. Dai M. (2015). Strategies and methods for the synthesis of anticancer natural product neopeltolide and its analogs. Curr. Org. Chem. 19 (10), 871–885. 10.2174/1385272819666150119225149 27182194 PMC4863658

[B7] Bauer K. Kazmaier U. (2023). Total synthesis of thiamyxins A–C and thiamyxin E, a potent class of RNA‐virus‐inhibiting (cyclo) depsipeptides. Angew. Chem. Int. Ed. 62, e202305445. 10.1002/anie.202305445 37256588

[B8] Bold C. P. Gut M. Schürmann J. Lucena‐Agell D. Gertsch J. Diaz J. F. (2021). Synthesis of morpholine‐based analogues of (−)‐Zampanolide and their biological activity. Chemistry–A Eur. J. 27 (19), 5936–5943. 10.1002/chem.202003996 33078440

[B9] Brütsch T. M. Cotter E. Lucena‐Agell D. Redondo‐Horcajo M. Davies C. Pfeiffer B. (2023). Synthesis and structure‐activity relationship studies of C (13)‐desmethylene‐(−)‐zampanolide analogs. Chemistry–A Eur. J. 29 (36), e202300703. 10.1002/chem.202300703 37057902

[B10] Chambers K. J. Sanghong P. Carter Martos D. Casoni G. Mykura R. C. Prasad Hari D. (2023). Stereospecific conversion of boronic esters into enones using methoxyallene: application in the total synthesis of 10‐deoxymethynolide. Angew. Chem. 135 (50), e202312054. 10.1002/anie.202312054 PMC10953306 37877778

[B11] Chandankar S. S. Raghavan S. (2020). Stereoselective synthesis of dysoxylactam A. Org. Lett. 22 (2), 653–655. 10.1021/acs.orglett.9b04426 31904983

[B137] Chandra J. Manne S. R. Mondal S. Mandal B. (2018). (E)-Ethyl-2-cyano-2-(((2, 4, 6-trichlorobenzoyl) oxy) imino) acetate: A Modified Yamaguchi Reagent for Enantioselective Esterification, Thioesterification, Amidation, and Peptide Synthesis. ACS omega 3 (6), 6120–6133. 10.1021/acsomega.8b00732 30023940 PMC6044346

[B12] Chang J.-L. Xu H.-Z. Zhou J. Zhou M. Zhang X. Guo Y. (2020). Antimicrobial furancarboxylic acids from a Penicillium sp. J. Nat. Prod. 83 (12), 3606–3613. 10.1021/acs.jnatprod.0c00758 33314934

[B13] Chen J. Jiang Y. Yan J. Xu C. Ye T. (2023). Total syntheses of colletopeptide A and colletotrichamide A. Molecules 28 (20), 7194. 10.3390/molecules28207194 37894673 PMC10608858

[B14] Chen Q.-H. Kingston D. G. (2014). Zampanolide and dactylolide: cytotoxic tubulin-assembly agents and promising anticancer leads. Nat. Product. Rep. 31 (9), 1202–1226. 10.1039/c4np00024b PMC4126874 24945566

[B15] Chlipala G. E. Tri P. H. Hung N. V. Krunic A. Shim S. H. Soejarto D. D. (2010). Nhatrangins A and B, aplysiatoxin-related metabolites from the marine cyanobacterium Lyngbya majuscula from Vietnam. J. Nat. Prod. 73 (4), 784–787. 10.1021/np100002q 20373744 PMC2977956

[B16] Decultot L. Clark J. S. (2022). Synthetic studies on amphidinolide F: exploration of macrocycle construction by intramolecular stille coupling. Org. Lett. 24 (41), 7600–7604. 10.1021/acs.orglett.2c03045 36223230 PMC9594353

[B17] Deng Y. Balunas M. J. Kim J.-A. Lantvit D. D. Chin Y.-W. Chai H. (2009). Bioactive 5, 6-dihydro-α-pyrone derivatives from Hyptis brevipes. J. Nat. Prod. 72 (6), 1165–1169. 10.1021/np9001724 19422206 PMC2883770

[B18] Depa M. R. Potla S. Narkhede U. C. Jadhav V. D. Sridhar G. Vidavalur S. (2021). Total synthesis of neocosmosin A. Synth. Commun. 51 (18), 2817–2823. 10.1080/00397911.2021.1952435

[B19] Dhimitruka I. SantaLucia J. (2006). Investigation of the Yamaguchi esterification mechanism. Synthesis of a Lux-S enzyme inhibitor using an improved esterification method. Org. Lett. 8 (1), 47–50. 10.1021/ol0524048 16381564

[B20] Dissanayake G. C. Ndi C. N. Markley J. L. Martinez J. B. Hanson P. R. (2023). Total synthesis of sanctolide A and formal synthesis of (2 S)-sanctolide A. J. Org. Chem. 88 (2), 805–817. 10.1021/acs.joc.2c01922 36602547 PMC10718183

[B21] Farlow M. R. Thompson R. E. Wei L.-J. Tuchman A. J. Grenier E. Crockford D. (2019). A randomized, double-blind, placebo-controlled, phase II study assessing safety, tolerability, and efficacy of bryostatin in the treatment of moderately severe to severe Alzheimer’s disease. J. Alzheimer's Dis. 67 (2), 555–570. 10.3233/jad-180759 30530975 PMC6398557

[B22] Feng L. Wang J. Liu S. Zhang X.-J. Bi Q.-R. Hu Y.-Y. (2019). Colletopeptides A–D, anti-inflammatory cyclic tridepsipeptides from the plant endophytic fungus Colletotrichum sp. S8. J. Nat. Prod. 82 (6), 1434–1441. 10.1021/acs.jnatprod.8b00829 31181925

[B23] Field J. J. Singh A. J. Kanakkanthara A. Halafihi T. i. Northcote P. T. Miller J. H. (2009). Microtubule-stabilizing activity of zampanolide, a potent macrolide isolated from the Tongan marine sponge Cacospongia mycofijiensis. J. Med. Chem. 52 (22), 7328–7332. 10.1021/jm901249g 19877653

[B24] Fritz L. Wienhold S. Hackl S. Bach T. (2022). Total synthesis of Pulvomycin D. Chemistry–A Eur. J. 28 (3), e202104064. 10.1002/chem.202104064 PMC9299864 34792826

[B25] Fujiki H. Tanaka Y. Miyake R. Kikkawa U. Nishizuka Y. Sugimura T. (1984). Activation of calcium-activated, phospholipid-dependent protein kinase (protein kinase C) by new classes of tumor promoters: teleocidin and debromoaplysiatoxin. Biochem. biophysical Res. Commun. 120 (2), 339–343. 10.1016/0006-291x(84)91259-2 6233970

[B26] Fuska J. Ivanitskaya L. Horakova K. Kuhr I. (1974). The cytotoxic effects of a new antibiotic vermiculine. J. Antibiotics 27 (2), 141–142. 10.7164/antibiotics.27.141 4856963

[B27] Fuska J. Nemec P. Kuhr I. (1972). Vermiculine, a new antiprotozoal antibiotic from Penicillium vermiculatum. J. Antibiotics 25 (4), 208–211. 10.7164/antibiotics.25.208 4559271

[B28] Gao J. Radwan M. M. León F. Dale O. R. Husni A. S. Wu Y. (2013). 'Neocosmospora sp.-derived resorcylic acid lactones with *in vitro* binding affinity for human opioid and cannabinoid receptors. J. Nat. Prod. 76 (5), 824–828. 10.1021/np300653d 23659286 PMC3723356

[B29] Ghosh A. K. Yuan H. (2022). Total syntheses of the proposed structure of iriomoteolide-1a,-1b and synthesis of three derivatives for structural studies. Mar. Drugs 20 (10), 587. 10.3390/md20100587 36286411 PMC9605196

[B30] Gillsch F. Mbui F. Bilitewski U. Schobert R. (2022a). Synthesis and bioactivity of thiocarboxylic A and derivatives. J. Nat. Prod. 85 (12), 2828–2835. 10.1021/acs.jnatprod.2c00870 36416745

[B31] Gillsch F. Zeng H. Bär S. I. Schrey H. Schobert R. (2022b). Synthesis and bioactivity of ophiofuranones A and B. J. Org. Chem. 87 (9), 6520–6523. 10.1021/acs.joc.2c00521 35471021

[B32] Gobin J. Moore C. H. Reeve Jr J. R. Wong D. K. Gibson B. W. Horwitz M. A. (1995). Iron acquisition by *Mycobacterium tuberculosis*: isolation and characterization of a family of iron-binding exochelins. Proc. Natl. Acad. Sci. 92 (11), 5189–5193. 10.1073/pnas.92.11.5189 7761471 PMC41874

[B33] Goda Y. Fuwa H. (2023). Total synthesis of (−)-Enigmazole B. Org. Lett. 25 (47), 8402–8407. 10.1021/acs.orglett.3c03002 37796572

[B34] Gutiérrez C. Serrano-Villar S. Madrid-Elena N. Pérez-Elías M. J. Martín M. E. Barbas C. (2016). Bryostatin-1 for latent virus reactivation in HIV-infected patients on antiretroviral therapy. Aids 30 (9), 1385–1392. 10.1097/qad.0000000000001064 26891037

[B35] Haack P. A. Harmrolfs K. Bader C. D. Garcia R. Gunesch A. P. Haid S. (2022). 'Thiamyxins: structure and biosynthesis of myxobacterial RNA‐virus inhibitors. Angew. Chem. Int. Ed. 61 (52), e202212946. 10.1002/anie.202212946 PMC10100342 36208117

[B36] Hamilton-Miller J. (1973). Chemistry and biology of the polyene macrolide antibiotics. Bacteriol. Rev. 37 (2), 166–196. 10.1128/mmbr.37.3.166-196.1973 PMC413810 4578757

[B37] Haslam E. (1980). Recent developments in methods for the esterification and protection of the carboxyl group. Tetrahedron 36 (17), 2409–2433. 10.1016/0040-4020(80)80219-5

[B38] Hegde V. R. Pu H. Patel M. Das P. R. Strizki J. Gullo V. P. (2004). Three new compounds from the plant Lippia alva as inhibitors of chemokine receptor 5 (CCR5). Bioorg. and Med. Chem. Lett. 14 (21), 5339–5342. 10.1016/j.bmcl.2004.08.021 15454223

[B39] Hellwig V. Mayer-Bartschmid A. Müller H. Greif G. Kleymann G. Zitzmann W. (2003). Pochonins A− F, new antiviral and antiparasitic resorcylic acid lactones from Pochonia c hlamydosporia var. c atenulata. J. Nat. Prod. 66 (6), 829–837. 10.1021/np020556v 12828470

[B40] Hikota M. Sakurai Y. Horita K. Yonemitsu O. (1990). Synthesis of erythronolide A via a very efficient macrolactonization under usual acylation conditions with the Yamaguchi reagent. Tetrahedron Lett. 31 (44), 6367–6370. 10.1016/s0040-4039(00)97066-7

[B41] Hopkins C. D. Wipf P. (2009). 'Isolation, biology and chemistry of the disorazoles: new anti-cancer macrodiolides. Nat. Product. Rep. 26 (5), 585–601. 10.1039/b813799b PMC2711774 19387496

[B42] Horáková K. Kernacova B. Nemec P. Fuska J. (1976). Characterization of the cytotoxic activity of vermiculine. J. Antibiotics 29 (10), 1109–1111. 10.7164/antibiotics.29.1109 994328

[B43] Horwitz L. D. Horwitz M. A. (2014). The exochelins of pathogenic mycobacteria: unique, highly potent, lipid-and water-soluble hexadentate iron chelators with multiple potential therapeutic uses. Antioxidants and redox Signal. 21 (16), 2246–2261. 10.1089/ars.2013.5789 PMC4224048 24684595

[B44] Inanaga J. Hirata K. Saeki H. Katsuki T. Yamaguchi M. (1979). A rapid esterification by means of mixed anhydride and its application to large-ring lactonization. Bull. Chem. Soc. Jpn. 52 (7), 1989–1993. 10.1246/bcsj.52.1989

[B45] Irschik H. Jansen R. Gerth K. Höfle G. Reichenbach H. (1995). Antibiotics from gilding bacteria. No.67. Sorangiolid A, a new antibiotic isolated from the myxobacterium Sorangium cellulosumSo ce 12. J. Antibiotics 48 (8), 886–887. 10.7164/antibiotics.48.886 7592035

[B46] Ishibashi M. Kobayashi J. i. (1997). Amphidinolides: unique macrolides from marine dinoflagellates. Heterocycles 1 (44), 543–572. 10.3987/rev-96-sr1

[B47] Jansen C. U. Nørskov A. Mortensen K. T. Qvortrup K. M. (2023). Convergent total synthesis of exochelin 772SM. J. Org. Chem. 88 (13), 8669–8673. 10.1021/acs.joc.3c00561 37294812

[B48] Jansen R. Irschik H. Meyer H. Reichenbach H. Wray V. Schomburg D. (1995). Antibiotics from gliding bacteria, LXIV. Isolation and structure elucidation of sorangiolides A and B, novel macrocyclic lactone carboxylic acids from *Sorangium cellulosum* . Liebigs Ann. 1995 (5), 867–872. 10.1002/jlac.1995199505125

[B49] Jansen R. (1994). Disorazoles, highly cytotoxic metabolites from the sorangicin-producing bacterium Sorangium cellulosum, strain So ce 12. Liebigs Ann. Chem. 759–773. 10.1002/jlac.199419940802

[B51] Jena B. K. Reddy G. S. Mohapatra D. K. (2017). First asymmetric total synthesis of aspergillide D. Org. and Biomol. Chem. 15 (8), 1863–1871. 10.1039/c6ob02435a 28165093

[B138] Jensen F. (1994). Introduction to computational chemistry. (John Wiley & Sons).

[B52] Jordan A. Whymark K. D. Sydenham J. Sneddon H. F. (2021). A solvent-reagent selection guide for Steglich-type esterification of carboxylic acids. Green Chem. 23 (17), 6405–6413. 10.1039/d1gc02251b

[B53] Joseph T. Sahoo S. Halligudi S. (2005). Brönsted acidic ionic liquids: a green, efficient and reusable catalyst system and reaction medium for Fischer esterification. J. Mol. Catal. A Chem. 234 (1-2), 107–110. 10.1016/j.molcata.2005.03.005

[B54] Kang H.-S. Krunic A. Orjala J. (2012). Sanctolide A, a 14-membered PK–NRP hybrid macrolide from the cultured cyanobacterium Oscillatoria sancta (SAG 74.79). Tetrahedron Lett. 53 (28), 3563–3567. 10.1016/j.tetlet.2012.04.136 22711943 PMC3375721

[B55] Kawahara T. Kagaya N. Masuda Y. Doi T. Izumikawa M. Ohta K. (2015). Foxo3a inhibitors of microbial origin, JBIR-141 and JBIR-142. Org. Lett. 17 (21), 5476–5479. 10.1021/acs.orglett.5b02842 26493489

[B56] Khan Z. Javed F. Shamair Z. Hafeez A. Fazal T. Aslam A. (2021). Current developments in esterification reaction: a review on process and parameters. J. Industrial Eng. Chem. 103, 80–101. 10.1016/j.jiec.2021.07.018

[B57] Kim G. Chu-Moyer M. Y. Danishefsky S. J. (1990). The total synthesis of dl-indolizomycin. J. Am. Chem. Soc. 112 (5), 2003–2005. 10.1021/ja00161a059

[B58] Kim J.-S. Lee I.-K. Kim D.-W. Yun B.-S. (2016). Aureosurfactin and 3-deoxyaureosurfactin, novel biosurfactants produced by Aureobasidium pullulans L3-GPY. J. Antibiotics 69 (10), 759–761. 10.1038/ja.2015.141 26758494

[B59] Kotammagari T. K. (2014). '2, 4, 6-trichlorobenzoyl chloride (Yamaguchi reagent). Synlett 25 (09), 1335–1336. 10.1055/s-0033-1341245

[B60] Kranzler M. Syrowatka M. Leitsch D. Winnips C. Walochnik J. (2015). Pentamycin shows high efficacy against Trichomonas vaginalis. Int. J. Antimicrob. agents 45 (4), 434–437. 10.1016/j.ijantimicag.2014.12.024 25703311

[B61] Kumari K. Syed T. Krishna A. Muvvala S. Nowduri A. Sridhar C. (2022). Steroselective total synthesis of Neocosmosin B. Tetrahedron 112, 132723. 10.1016/j.tet.2022.132723

[B62] Kumari K. Syed T. Krishna A. Muvvala S. Nowduri A. Sridhar C. (2023). Stereoselective total synthesis of Aspergillide D. Nat. Prod. Res. 37 (20), 3402–3408. 10.1080/14786419.2022.2078321 35666807

[B63] Kumar Palli K. Reddy Anugu R. Chandrasekhar S. (2021). Total synthesis of (−)‐4‐epi‐Englerin A. Eur. J. Org. Chem. 2021 (22), 3190–3196. 10.1002/ejoc.202100354

[B64] Lai Y. Dai W. M. (2021). Modular total synthesis of (–)‐Palmyrolide A and (+)‐(S,S)‐Palmyrolide A via ring‐closing metathesis and alkene isomerization†. Chin. J. Chem. 39 (1), 69–74. 10.1002/cjoc.202000458

[B65] Li Y.-L. Zhu R.-X. Li G. Wang N.-N. Liu C.-Y. Zhao Z.-T. (2019). Secondary metabolites from the endolichenic fungus Ophiosphaerella korrae. RSC Adv. 9 (8), 4140–4149. 10.1039/c8ra10329a 35520149 PMC9060614

[B66] Liu C.-P. Xie C.-Y. Zhao J.-X. Ji K.-L. Lei X.-X. Sun H. (2019). Dysoxylactam a: a macrocyclolipopeptide reverses p-glycoprotein-mediated multidrug resistance in cancer cells. J. Am. Chem. Soc. 141 (17), 6812–6816. 10.1021/jacs.9b02259 30998329

[B67] Liu H. Ottosen R. N. Jennet K. M. Svenningsen E. B. Kristensen T. F. Biltoft M. (2021). Macrodiolide diversification reveals broad immunosuppressive activity that impairs the cGAS‐STING pathway. Angew. Chem. Int. Ed. 60 (34), 18734–18741. 10.1002/anie.202105793 34124819

[B68] Lizzadro L. Spieß O. Collisi W. Stadler M. Schinzer D. (2022). Extending the structure‐activity relationship of disorazole C1: exchanging the oxazole ring by thiazole and influence of chiral centers within the disorazole core on cytotoxicity. ChemBioChem 23 (20), e202200458. 10.1002/cbic.202200458 35998215 PMC9826379

[B69] Lizzadro L. Spieß O. Schinzer D. (2021). Total synthesis of (−)-disorazole C1. Org. Lett. 23 (12), 4543–4547. 10.1021/acs.orglett.1c01123 34037403

[B70] Majhi S. (2021). Applications of Yamaguchi method to esterification and macrolactonization in total synthesis of bioactive natural products. ChemistrySelect 6 (17), 4178–4206. 10.1002/slct.202100206

[B71] Maram L. Das B. (2015). A stereoselective total synthesis of xyolide, a natural bioactive nonenolide. Helvetica Chim. Acta 98 (5), 674–682. 10.1002/hlca.201400291

[B72] Matthew S. Salvador L. A. Schupp P. J. Paul V. J. Luesch H. (2010). Cytotoxic halogenated macrolides and modified peptides from the apratoxin-producing marine cyanobacterium Lyngbya bouillonii from Guam. J. Nat. Prod. 73 (9), 1544–1552. 10.1021/np1004032 20704304 PMC2965600

[B73] Meyer C. C. Verboom K. L. Evarts M. M. Jung W.-O. Krische M. J. (2023). Allyl alcohol as an acrolein equivalent in enantioselective C–C coupling: total synthesis of amphidinolides R, J, and S. J. Am. Chem. Soc. 145 (14), 8242–8247. 10.1021/jacs.3c01809 36996284 PMC10101927

[B74] Michel K. Demarco P. Nagarajan R. (1977). The isolation and structure elucidation of macrocyclic lactone antibiotic, A26771B. J. antibiotics 30 (7), 571–575. 10.7164/antibiotics.30.571 142754

[B75] Mittendorf F. Quambusch M. Kirsch S. F. (2023). Total synthesis of both enantiomers of the biosurfactant aureosurfactin via bidirectional synthesis with a chiral Horner–Wittig building block. Org. and Biomol. Chem. 21 (22), 4574–4577. 10.1039/d3ob00650f 37222559

[B76] Molawi K. Delpont N. Echavarren A. M. (2010). Enantioselective synthesis of (−)‐englerins A and B. Angew. Chem. 20 (122), 3595–3597. 10.1002/anie.201000890 20544903

[B77] Moon K. Cui J. Kim E. Riandi E. S. Park S. H. Byun W. S. (2020). Structures and biosynthetic pathway of pulvomycins B–D: 22-membered macrolides from an estuarine Streptomyces sp. Org. Lett. 22 (14), 5358–5362. 10.1021/acs.orglett.0c01249 32628027

[B78] Morishita M. Hada K. Kita M. Nishikawa T. (2023). The asymmetric total synthesis and configuration confirmation of aplysiaenal and nhatrangin A, truncated derivatives of aplysiatoxin and oscillatoxin. J. Nat. Prod. 86 (4), 1033–1041. 10.1021/acs.jnatprod.3c00077 36999535

[B79] Mukhopadhyay T. K. Trauner D. (2022). Concise synthesis of glycerophospholipids. J. Org. Chem. 88 (15), 11253–11257. 10.1021/acs.joc.2c02096 36449029

[B80] Mulligan C. N. (2005). Environmental applications for biosurfactants. Environ. Pollut. 133 (2), 183–198. 10.1016/j.envpol.2004.06.009 15519450

[B81] Munawar S. Zahoor A. F. Ali S. Javed S. Irfan M. Irfan A. (2022). Mitsunobu reaction: a powerful tool for the synthesis of natural products: a review. Molecules 27 (20), 6953. 10.3390/molecules27206953 36296545 PMC9609662

[B82] Munawar S. Zahoor A. F. Hussain S. M. Ahmad S. Mansha A. Parveen B. (2024). Steglich esterification: a versatile synthetic approach toward the synthesis of natural products, their analogues/derivatives. Heliyon 10 (1), e23416. 10.1016/j.heliyon.2023.e23416 38170008 PMC10758822

[B83] Munir R. Zahoor A. F. Nazeer U. Saeed M. A. Mansha A. Irfan A. (2023). Gilman reagent toward the synthesis of natural products. RSC Adv. 13 (50), 35172–35208. 10.1039/d3ra07359a 38053693 PMC10694855

[B84] Nakazato K. Oda M. Fuwa H. (2022). Total synthesis of (+)-neopeltolide by the macrocyclization/transannular pyran cyclization strategy. Org. Lett. 24 (22), 4003–4008. 10.1021/acs.orglett.2c01429 35649194

[B85] Nishio Y. Kawazu A. Hirano S. Matsubara H. (2016). Preparation of fluorous Yamaguchi reagents and evaluation of their reactivity in esterification. Tetrahedron 72 (5), 720–725. 10.1016/j.tet.2015.12.026

[B86] Oh D.-C. Jensen P. R. Fenical W. (2006). Zygosporamide, a cytotoxic cyclic depsipeptide from the marine-derived fungus Zygosporium masonii. Tetrahedron Lett. 47 (48), 8625–8628. 10.1016/j.tetlet.2006.08.113

[B87] Ohno O. Ikeda Y. Sawa R. Igarashi M. Kinoshita N. Suzuki Y. (2004). 'Isolation of heptadepsin, a novel bacterial cyclic depsipeptide that inhibits lipopolysaccharide activity. Chem. and Biol. 11 (8), 1059–1070. 10.1016/j.chembiol.2004.05.016 15324807

[B88] Oku N. Takada K. Fuller R. W. Wilson J. A. Peach M. L. Pannell L. K. (2010). Isolation, structural elucidation, and absolute stereochemistry of enigmazole A, a cytotoxic phosphomacrolide from the Papua New Guinea marine sponge Cinachyrella enigmatica. J. Am. Chem. Soc. 132 (30), 10278–10285. 10.1021/ja1016766 20590096 PMC3850515

[B89] Okuno Y. Isomura S. Nishibayashi A. Hosoi A. Fukuyama K. Ohba M. (2014). Modified Yamaguchi reagent: convenient and efficient esterification. Synth. Commun. 44 (19), 2854–2860. 10.1080/00397911.2014.919659

[B90] Park J. Ngo H. V. Jin H.-E. Lee K. W. Lee B.-J. (2022). Hydroxyl group-targeted conjugate and its self-assembled nanoparticle of peptide drug: effect of degree of saturation of fatty acids and modification of physicochemical properties. Int. J. Nanomedicine Vol. 17, 2243–2260. 10.2147/ijn.s356804 PMC9124699 35615542

[B91] Patocka J. Soukup O. Kuca K. (2013). Resorcylic acid lactones as the protein kinase inhibitors, naturally occuring toxins. Mini Rev. Med. Chem. 13 (13), 1873–1878. 10.2174/13895575113136660096 24070207

[B92] Payero T. D. Vicente C. M. Rumbero Á. Barreales E. G. Santos-Aberturas J. de Pedro A. (2015). Functional analysis of filipin tailoring genes from Streptomyces filipinensis reveals alternative routes in filipin III biosynthesis and yields bioactive derivatives. Microb. Cell Factories 14, 114–14. 10.1186/s12934-015-0307-4 PMC4527110 26246267

[B93] Pereira A. R. Cao Z. Engene N. Soria-Mercado I. E. Murray T. F. Gerwick W. H. (2010). Palmyrolide A, an unusually stabilized neuroactive macrolide from Palmyra Atoll cyanobacteria. Org. Lett. 12 (20), 4490–4493. 10.1021/ol101752n 20845912 PMC2965064

[B94] Pérez-Moreno G. Cantizani J. Sánchez-Carrasco P. Ruiz-Pérez L. M. Martín J. El Aouad N. (2016). Discovery of new compounds active against Plasmodium falciparum by high throughput screening of microbial natural products. PLoS One 11 (1), e0145812. 10.1371/journal.pone.0145812 26735308 PMC4703298

[B95] Pirovani R. V. Brito G. A. Barcelos R. C. Pilli R. A. (2015). Enantioselective total synthesis of (+)-lyngbyabellin M. Mar. drugs 13 (6), 3309–3324. 10.3390/md13063309 26023838 PMC4483630

[B96] Pujari S. A. Gowrisankar P. Kaliappan K. P. (2011). A shimizu non‐aldol approach to the formal total synthesis of palmerolide A. Chemistry–An Asian J. 6 (11), 3137–3151. 10.1002/asia.201100429 21913332

[B97] Radha Krishna M. Sridhar G. Syed T. Jayaprakash H. V. (2022). Stereoselective total synthesis of (-)-curvularin. Synth. Commun. 52 (1), 37–42. 10.1080/00397911.2021.1979043

[B98] Ratnayake R. Covell D. Ransom T. T. Gustafson K. R. Beutler J. A. (2009). Englerin A, a selective inhibitor of renal cancer cell growth, from Phyllanthus engleri. Org. Lett. 11 (1), 57–60. 10.1021/ol802339w 19061394 PMC2651161

[B99] Reddy G. N. Gudisela M. R. Boddiboyena R. Prasad K. (2022). Total synthesis of sumalactone A. Synth. Commun. 52 (17), 1713–1720. 10.1080/00397911.2022.2111527

[B100] Saha S. Paul D. Goswami R. K. (2020). Cyclodepsipeptide alveolaride C: total synthesis and structural assignment. Chem. Sci. 11 (41), 11259–11265. 10.1039/d0sc04478d 34094366 PMC8162944

[B101] Sahana M. H. Paul D. Sharma H. Goswami R. K. (2023). Total synthesis of antibacterial macrolide sorangiolide A. Org. Lett. 25 (43), 7827–7831. 10.1021/acs.orglett.3c03066 37856450

[B102] Sahana M. H. Saha D. Goswami R. K. (2022). Total synthesis of strasseriolide A. J. Org. Chem. 87 (17), 11805–11815. 10.1021/acs.joc.2c01595 35960823

[B103] Sakai T. Sameshima T. Matsufuji M. Kawamura N. Dobashi K. Mizui Y. (2004). 'Pladienolides, new substances from culture of Streptomyces platensis Mer-11107 I. Taxonomy, fermentation, isolation and screening. J. antibiotics 57 (3), 173–179. 10.7164/antibiotics.57.173 15152802

[B104] Salituro L. J. Pazienza J. E. Rychnovsky S. D. (2022). Total syntheses of strasseriolide A and B, antimalarial macrolide natural products. Org. Lett. 24 (5), 1190–1194. 10.1021/acs.orglett.1c04340 35094508

[B105] Schmidt F. Viswanathan Ammanath A. Götz F. Maier M. E. (2023). Synthesis of berkeleylactone A by ring‐closing alkyne metathesis. Eur. J. Org. Chem. 26 (36), e202300615. 10.1002/ejoc.202300615

[B106] Shahzadi I. Zahoor A. F. Tüzün B. Mansha A. Anjum M. N. Rasul A. (2022). Repositioning of acefylline as anti-cancer drug: synthesis, anticancer and computational studies of azomethines derived from acefylline tethered 4-amino-3-mercapto-1, 2, 4-triazole. Plos one 17 (12), e0278027. 10.1371/journal.pone.0278027 36520942 PMC9754256

[B107] Shimbo K. Tsuda M. Izui N. Kobayashi J. i. (2002). Amphidinolide W, a new 12-membered macrolide from dinoflagellate Amphidinium sp. J. Org. Chem. 67 (3), 1020–1023. 10.1021/jo016089o 11856056

[B108] Shotwell J. B. Roush W. R. (2004). Synthesis of the C11− C29 fragment of amphidinolide F. Org. Lett. 6 (21), 3865–3868. 10.1021/ol048381z 15469369

[B109] Singh A. Van Hamme J. D. Ward O. P. (2007). Surfactants in microbiology and biotechnology: Part 2. Application aspects. Biotechnol. Adv. 25 (1), 99–121. 10.1016/j.biotechadv.2006.10.004 17156965

[B110] Stierle A. A. Stierle D. B. Decato D. Priestley N. D. Alverson J. B. Hoody J. (2017). The berkeleylactones, antibiotic macrolides from fungal coculture. J. Nat. Prod. 80 (4), 1150–1160. 10.1021/acs.jnatprod.7b00133 28326781 PMC5467647

[B111] Takada K. Oku N. Peach M. L. Ransom T. T. Henrich C. J. Gustafson K. R. (2023). Enigmazole phosphomacrolides from the marine sponge Cinachyrella enigmatica. J. Org. Chem. 88 (15), 10996–11002. 10.1021/acs.joc.3c00963 37471139

[B112] Tan L. T. (2007). Bioactive natural products from marine cyanobacteria for drug discovery. Phytochemistry 68 (7), 954–979. 10.1016/j.phytochem.2007.01.012 17336349

[B113] Tanaka J.-i. Higa T. (1996). 'Zampanolide, a new cytotoxic marcrolide from a marine sponge. Tetrahedron Lett. 37 (31), 5535–5538. 10.1016/0040-4039(96)01149-5

[B114] Tsuda M. Oguchi K. Iwamoto R. Okamoto Y. Fukushi E. Kawabata J. (2007a). Iriomoteolides-1b and-1c, 20-membered macrolides from a marine dinoflagellate Amphidinium species. J. Nat. Prod. 70 (10), 1661–1663. 10.1021/np0702537 17927263

[B115] Tsuda M. Oguchi K. Iwamoto R. Okamoto Y. Kobayashi J. i. Fukushi E. (2007b). Iriomoteolide-1a, a potent cytotoxic 20-membered macrolide from a benthic dinoflagellate Amphidinium species. J. Org. Chem. 72 (12), 4469–4474. 10.1021/jo070414b 17500570

[B116] Ulanovskaya O. A. Janjic J. Suzuki M. Sabharwal S. S. Schumacker P. T. Kron S. J. (2008). Synthesis enables identification of the cellular target of leucascandrolide A and neopeltolide. Nat. Chem. Biol. 4 (7), 418–424. 10.1038/nchembio.94 18516048 PMC2673112

[B117] Umaña C. A. Henry J. L. Saltzman C. T. Sackett D. L. Jenkins L. M. Taylor R. E. (2023). Linear (−)‐Zampanolide: flexibility in conformation–activity relationships. ChemMedChem 18 (19), e202300292. 10.1002/cmdc.202300292 37552215 PMC10615712

[B118] Vakiti A. R. Valluru K. R. Verma S. N. Syed T. Sridhar G. Kumar J. S. (2022). Total synthesis of berkeleylactone F. Synth. Commun. 52 (2), 205–211. 10.1080/00397911.2021.2012804

[B119] Valeev R. Sunagatullina G. Miftakhov M. (2019). Yamaguchi esterification in the synthetic approaches to precursors of epothilone D analogs. Russ. J. Org. Chem. 55, 1439–1441. 10.1134/s1070428019090264

[B120] Villa R. Mandel A. L. Jones B. D. La Clair J. J. Burkart M. D. (2012). Structure of FD-895 revealed through total synthesis. Org. Lett. 14 (21), 5396–5399. 10.1021/ol3023006 23072504 PMC3518397

[B121] Wei Y. Shi M. (2013). Recent advances in organocatalytic asymmetric morita–baylis–hillman/aza-morita–baylis–hillman reactions. Chem. Rev. 113 (8), 6659–6690. 10.1021/cr300192h 23679920

[B122] Wender P. A. Luu-Nguyen Q. H. Sloane J. L. Ranjan A. (2022). Trimethylene methane dianion equivalent for the asymmetric consecutive allylation of aldehydes: applications to prins-driven macrocyclizations for the synthesis of bryostatin 1 and analogues. J. Org. Chem. 87 (23), 15925–15937. 10.1021/acs.joc.2c02047 36378802 PMC13186262

[B123] Willot M. Radtke L. Könning D. Fröhlich R. Gessner V. Strohmann C. (2010). Synthesis of engerlin A. Synfacts 2010 (03), 0262. 10.1055/s-0029-1219246

[B124] Wittmann L. Wachter A. C. Schrey H. Schobert R. (2022). Synthetic approach to the natural N-nitrosohydroxylamino tetramic acid JBIR-141. Org. Lett. 24 (28), 5171–5175. 10.1021/acs.orglett.2c02006 35815821

[B125] Wright A. E. Botelho J. C. Guzmán E. Harmody D. Linley P. McCarthy P. J. (2007). Neopeltolide, a macrolide from a lithistid sponge of the family Neopeltidae. J. Nat. Prod. 70 (3), 412–416. 10.1021/np060597h 17309301

[B126] Wu J.-Z. Wang Z. Qiao C. (2012). Synthesis of stagonolide C from mulzer epoxide. Tetrahedron Lett. 53 (9), 1153–1155. 10.1016/j.tetlet.2011.12.102

[B127] Wu Y.-H. Zhang Z.-H. Zhong Y. Huang J.-J. Li X.-X. Jiang J.-Y. (2017). Sumalactones A–D, four new curvularin-type macrolides from a marine deep sea fungus Penicillium Sumatrense. RSC Adv. 7 (63), 40015–40019. 10.1039/c7ra06933b

[B128] Yamamoto H. Muramatsu W. (2019). Modified Yamaguchi reagent mediated coupling reactions. Synfacts 15 (01), 0100. 10.1055/s-0037-1611429

[B129] Yang G. Z. Wang L. Fan Y. Y. Lai Z. W. Yu X. N. Lou L. G. (2022). Concise total synthesis of dysoxylactam A and a simplified analog. Chin. J. Chem. 40 (17), 2027–2034. 10.1002/cjoc.202200123

[B130] Yasoshima K. Yoshida M. Doi T. (2022). The concise synthesis of a tert-butoxycarbonyl derivative of (3 R, 4 S)-4-Amino-3-hydroxy-7-(N-nitrosohydroxyamino)-2, 2-dimethylheptanoate, a component of JBIR-141. Bull. Chem. Soc. Jpn. 95 (5), 830–832. 10.1246/bcsj.20220035

[B131] Yoo M. Krische M. J. (2021). Total synthesis of the spliceosome modulator pladienolide B via asymmetric alcohol‐mediated syn‐and anti‐diastereoselective carbonyl crotylation. Angew. Chem. 133 (25), 14042–14047. 10.1002/ange.202103845 PMC8190871 33794050

[B132] Zhan J. Gunatilaka A. L. (2005). Microbial transformation of curvularin. J. Nat. Prod. 68 (8), 1271–1273. 10.1021/np0580309 16124776

[B133] Zhang Y. Liu Y. Du Y. (2021). First total synthesis of 27-deoxylyngbyabellin A. Synthesis 53 (16), 2874–2880. 10.1055/a-1478-9088

[B134] Zhao Z. Lv Q. Geng J. Liu Y. Hu C. Du Y. (2023). Stereoselective total synthesis of (+)-brevipolide H from D-galactal. Synthesis 55 (02), 341–346. 10.1055/a-1700-3520

[B135] Zief M. Woodside R. Schmitz H. (1957). Pulvomycin. Antibiotics and Chemother. 7 (7), 384–386. 24544485

[B136] Zulqurnain M. Aijijiyah N. P. Wati F. A. Fadlan A. Azminah A. Santoso M. (2023). Synthesis, *Mycobacterium tuberculosis* H37Rv inhibitory activity, and molecular docking study of pyrazinamide analogs. J. Appl. Pharm. Sci. 13 (11), 170–177. 10.7324/japs.2023.140149

