# Supplementary material for: Yamaguchi esterification: a key step toward the synthesis of natural products and their analogs—a review
Source: Front Chem. 2024 Oct 11;12:1477764. doi: 10.3389/fchem.2024.1477764 (PMC11503016; doi:10.3389/fchem.2024.1477764)
Supplement: Supplementary file 1 [file DataSheet1.docx]

**Supplementary file**

**Yamaguchi Esterification: A Key Step Toward The Synthesis of Natural Products And Their Analogues: A Review**

**Figure 1.** Structure of fluorous Yamaguchi reagent

**Figure 2.** Some previously reported biologically active natural products, synthesized via the employment of the Yamaguchi reagent.

**Scheme 1.** Synthesis of pulvomycin D.

**Figure 3**. (−)-Zampanolide analogues.

**Scheme 2.** Synthesis of precursors towards the synthesis of disorazole C_1_.

**Figure 4.** Analogues of disorazole C_1_.

**Scheme 3**. Synthesis of ophiofuranones A, ophiofuranones B, thiocarboxylic acid, and its three analogues.

**Scheme 4**. Synthesis of thiamyxin A, B, E & C.

**Scheme 5.** Synthesis of brevipolide H, (+)-aureosurfactin & (−)-aureosurfactin.
